# Prognostic role of macrophages and mast cells in the microenvironment of hepatocellular carcinoma after resection

**DOI:** 10.1186/s12885-024-11904-8

**Published:** 2024-01-29

**Authors:** Esraa Ali, Lenka Červenková, Richard Pálek, Filip Ambrozkiewicz, Petr Hošek, Ondrej Daum, Václav Liška, Kari Hemminki, Andriy Trailin

**Affiliations:** 1 Laboratory of Translational Cancer Genomics, Biomedical Center, Faculty of Medicine in Pilsen, Charles University, Alej Svobody 1655/76, Pilsen, 32300 Czech Republic; 2 Laboratory of Cancer Treatment and Tissue Regeneration, Biomedical Center, Faculty of Medicine in Pilsen, Charles University, Alej Svobody 1655/76, Pilsen, 32300 Czech Republic; 3 Department of Pathology, Third Faculty of Medicine, Charles University, Ruská 87, Prague, 10000 Czech Republic; 4 Department of Surgery and Biomedical Center, Faculty of Medicine in Pilsen, Charles University, Alej Svobody 80, Pilsen, 32300 Czech Republic; 5 Sikl’s Institute of Pathology, Faculty of Medicine and Teaching Hospital in Plzen, Charles University, Edvarda Beneše 13, Pilsen, 30599 Czech Republic; 6 Bioptická Laboratoř s.r.o, Mikulášské Nám. 4, Pilsen, 32600 Czech Republic; 7 Department of Cancer Epidemiology, German Cancer Research Center, Im Neuenheimer Feld 280, 69120 Heidelberg, Germany

**Keywords:** Hepatocellular carcinoma, Tumor-infiltrating macrophages, Mast cells, Inner margin, Peritumor area, Disease-free survival

## Abstract

**Background:**

The prognostic significance of mast cells and different phenotypes of macrophages in the microenvironment of hepatocellular carcinoma (HCC) following resection is unclear. We aimed in this study to assess the local distribution of infiltrating macrophages and mast cells of specific phenotypes in tissues of HCC and to evaluate their prognostic values for survival of post-surgical patients.

**Methods:**

The clinicopathological and follow-up data of 70 patients with HCC, who underwent curative resection of tumor from 1997 to 2019, were collected. The infiltration of CD68+ and CD163+ macrophages and CD117+ mast cells was assessed immunohistochemically in representative resected specimens of HCC and adjacent tissues. The area fraction (AF) of positively stained cells was estimated automatically using QuPath image analysis software in several regions, such as tumor center (TC), inner margin (IM), outer margin (OM), and peritumor (PT) area. The prognostic significance of immune cells, individually and in associations, for time to recurrence (TTR), disease-free survival (DFS), and overall survival (OS) was evaluated using Kaplan-Meier and Cox regression analyses.

**Results:**

High AF of CD68+ macrophages in TC and IM and high AF of mast cells in IM and PT area were associated with a longer DFS. High AF of CD163+ macrophages in PT area correlated with a shorter DFS. Patients from CD163TC^high^ & CD68TC^low^ group had a shorter DFS compared to all the rest of the groups, and cases with CD163IM^low^ & CD68IM^high^ demonstrated significantly longer DFS compared to low AF of both markers. Patients from CD68IM^high^ & CD163PT^low^ group, CD117IM^high^ & CD163PT^low^ group, and CD117PT^high^ & CD163PT^low^ group had a significantly longer DFS compared to all other combinations of respective cells.

**Conclusions:**

The individual prognostic impact of CD68+ and CD163+ macrophages and mast cells in the microenvironment of HCC after resection depends on their abundance and location, whereas the cumulative impact is built upon combination of different cell phenotypes within and between regions.

**Supplementary Information:**

The online version contains supplementary material available at 10.1186/s12885-024-11904-8.

## Background

Liver cancer, which in 75–85% of cases is represented by hepatocellular carcinoma (HCC), ranks as the third leading cause of cancer death worldwide [1]. The type, density, and location of adaptive immune cells within the tumor microenvironment (TME) have been reported to improve the estimation of HCC prognosis with the contribution of the commonly used tools of tumor-node-metastasis (TNM) staging, microvascular invasion, tumor multiplicity, and serum α-fetoprotein level [2]. Innate immune cells in TME could also improve the prediction of clinical outcomes in different cancers [3]. Some populations of tumor-associated innate immune cells, e.g. neutrophils, have been extensively evaluated in several HCC studies, which provided rather conclusive results as for their poor prognostic significance [4]. On the other hand opposite results were frequently reported on the prognostic values of tumor-associated macrophages (TAM) and mast cells in HCC [5–12]. TAM and mast cells are both innate immune cells with the capacity of modifying anti-tumor immune response from antigen-presentation to killing of tumor cells [13, 14]. Doing so they could orchestrate and regulate tumor initiation and progression [15].

TAM are highly plastic cells, which in HCC encompass resident Kupffer cells and monocyte-derived macrophages. All of these may have different functional states: from antitumor proinflammatory M1 (CD68+ CD163-) to protumor antiinflammatory M2 (CD68+ CD163+) macrophages [16], which may explain the discrepancies in reported effects of TAM in HCC. In addition, intratumor or peritumor localization of TAM seems to have an impact on their properties [17, 18].

Mast cells in the TME also can suppress as well as support cancer development and progression [19]. They can support pro-inflammatory pathways that could lead to the impairment of tumor progression [20]. On the other hand, they contribute to angiogenesis, facilitate metastasis and recruit TAM for pro-tumor response [14, 21]. Activated intratumor mast cells recruited TAM in gastric cancer murine model, resulting in increased tumor growth [22]. However, there are no published results on the predictive power of their concomitant infiltration in HCC tissues. Furthermore, overwhelming majorities of studies on mast cells and TAM have focused on viral hepatitis-associated HCC in Asian populations [6, 7, 23–25]. In this study, we aimed at analyzing the association of intratumoral and peritumoral TAM using a pan-macrophage marker CD68+ and their pro-tumor CD163+ subset, as well as CD117+ mast cells, individually and in pairs, with outcomes and clinicopathological characteristics of European patients after curative resection of HCC.

## Materials and methods

### Patients

We conducted a single-center, retrospective cohort study of 70 consecutive patients with pathologically confirmed stage I-IV HCC, who underwent curative-intent resection at Pilsen University Hospital between 1997 and 2019. Two of them were operated on for recurrent HCC. Pathology reports were reviewed. None of the patients had distant metastases nor had received neoadjuvant therapy such as chemotherapy or radiotherapy prior to surgery. We excluded three patients with poor-quality histological specimens and the remaining 67 patients were included into the study (Additional file 1: Table S1). This retrospective study was conducted in accordance with the ethical standards set out in the Declaration of Helsinki (2013 version); it was approved by the Ethics Committee of the Faculty of Medicine and the University Hospital in Pilsen (118/2021, 11 March 2021). The clinical stage of the tumor was determined according to the 8th edition of the American Joint Commission on Cancer [26]. The follow-up after resection was based on repeated imaging (computed tomography or magnetic resonance) in combination with serum α-fetoprotein levels at least two times per year. The exact timing of imaging was dependent on the clinician’s decision. Postoperative treatment was carried out in accordance with generally accepted recommendations. Among 29 patients, who experienced a tumor recurrence, 8 were reoperated, 8 received chemotherapy only, 5 received chemotherapy after reoperation, and 8 patients had missing data for postoperative treatment.

### Pathology and immunohistology

For each patient, 2 to 3 blocks of formalin-fixed paraffin-embedded tissue containing the center of the tumor, invasive margin, and peritumor area were retrieved from the pathology archive. Hematoxylin and eosin and Masson’s trichrome stained sections were used to evaluate the histopathological features of tumor. The Edmondson-Steiner and WHO grading systems were used to evaluate tumor differentiation (Additional file 1: Table S2) [27]. Architectural, nuclear, and nucleolar grades were also scored [27]. Patterns of invasion [28], micronodularity, presence of microvascular invasion, microsatellites, and necrosis were recorded as well. Assessment of the stromal component inside the tumor and the extent of encapsulation were based on a semiquantitative scale (0–3) [29]. One or two tissue sections of 4-µm thickness were cut and mounted onto BOND Plus Microscope Slides (Leica Biosystems Newcastle Ltd., Newcastle, UK). Immunoperoxidase detection of CD68+ and CD163+ macrophages, and CD117+ mast cells was performed using a BOND-III IHC/ISH autostainer. Monoclonal primary antibodies to CD68, CD163 and CD117 were used (Additional file 1: Table S3). After counterstaining with Mayer’s hematoxylin, the sections were embedded in the Micromount mounting medium (Leica Biosystems Newcastle Ltd., UK). Appropriate negative tissue control samples and positive (tonsils) were used throughout.

### Image analysis

Whole-slide scans were obtained using ZEISS AxioScan.Z1 scanner (Carl Zeiss Microscopy GmbH, Jena, Germany). An open-source software for image analysis (QuPath v.0.3.2) was used to make the estimation more objective, reliable and reproducible [30]. We used tools of QuPath to draw a border separating the malignant cell nests and adjacent non-tumor tissue (Fig. S1). When defining ROIs, we followed the recommendations of the International Immunooncology Biomarkers Working Group [31]. Inner margin (IM) and outer margin (OM) were extended automatically as 500 μm-wide regions on each side of the border [2]. The tumor center (TC) represents the remaining tumor area. The peritumor (PT) area was defined as the 500 μm-wide region adjacent to the OM [2]. The area fraction (AF) of positively stained cells was then determined automatically, as precise counting of irregularly shaped immune cells like macrophages can prove difficult [32]. Before analysis, color deconvolution was applied using the “Estimate Stain Vectors” command to produce normalized staining for each slide. Assessment of AF was performed using the “Pixel Classification” tool. After selecting a diaminobenzidine channel we set up a threshold, which enables optimal classification of negatively vs. positively stained pixels. To eliminate skewness in the distribution, we converted the raw AF data into corresponding percentile values and categorized them into low (below 25th percentile) vs. high (25th-100th percentile).

### Outcome measures and statistical methods

The primary endpoint was disease-free survival (DFS) that was considered as the time from tumor resection to the date of diagnosis of recurrence/metastasis or death from any cause. Secondary outcomes were time to recurrence (TTR), and overall survival (OS). TTR was considered as the time from the date of operation to the date of diagnosis of recurrence/metastasis. If relapse was not diagnosed, patients were censored either at the date of death or date of last follow-up. The proportion of patients without recurrence was defined as the recurrence-free proportion. OS was defined as the time from operation to death from any cause. Patients without relapse or death were censored at their last follow-up.

Continuous non-normally distributed data are expressed as median (min-max); their comparison was made either by Mann-Whitney U-test or by Friedman ANOVA, followed by Wilcoxon matched pairs test with Bonferroni correction. Proportions are expressed as raw data (percentages). The associations between pairs of ordinal or quantitative variables were assessed using Spearman correlation due to nonparametric distribution of most of the variables. DFS, TTR and OS were estimated using the Kaplan-Meier method and compared between groups with the log-rank test. To determine the prognostic value of individual predictors for TTR, DFS and OS, a univariable followed by multivariable Cox regression analysis was performed. As the development of HCC in the majority of patients was not associated with preceding viral hepatitis, we performed Cox analysis in the whole cohort and in the cohort of 58 patients with non-viral etiology of the HCC. Hazard ratios (HRs) showing the relative risk for high group compared with 1 for the low group, were calculated. GraphPad Prism 9.0 (GraphPad Software LLC) and Statistica 12 (StatSoft, Inc., Tulsa, OK, USA) were used for the statistical analyses. A 2-sided p value < 0.05 was considered statistically significant. The false discovery rate (FDR) was controlled using the Benjamini-Hochberg procedure based on the results of all significance tests performed within the study. At the baseline significance level of 0.05, the estimated FDR is 22%, indicating ca. 78% of the presented significant results to be true positives. A conservative overall FDR of 5% would require an individual significance level of 0.0047.

## Results

### Demographics of HCC patients

The demographics and clinical characteristics of the patients are shown in Additional file 1: Table S1. The patient’s median diagnostic age was 69 years, and males accounted for 77.6%. In terms of the cause of HCC, the most common background disease was non-alcoholic steatohepatitis (NASH) (23.9%). Most patients (67.8%) were of TNM stage I. Pathological characteristics of the resected tumors are listed in Additional file 1: Table S2. The majority of the tumors (70.1%) were histologically graded as G2 according to the Edmondson-Steiner method. Most prevalent growth types were mixed and desmoplastic.

### Outcomes

At the last follow-up, 29 (41.8%) patients had tumor recurrence (of which 89.7% patients had local recurrence), and 38 (56.7%) patients had died. At five years after surgery DFS and OS were 33.2% and 49.4%, respectively; recurrence-free survival proportion was 48.7% (Additional file 1: Table S4).

### Distribution of immune cells in different regions of interest

**Fig. 1:**
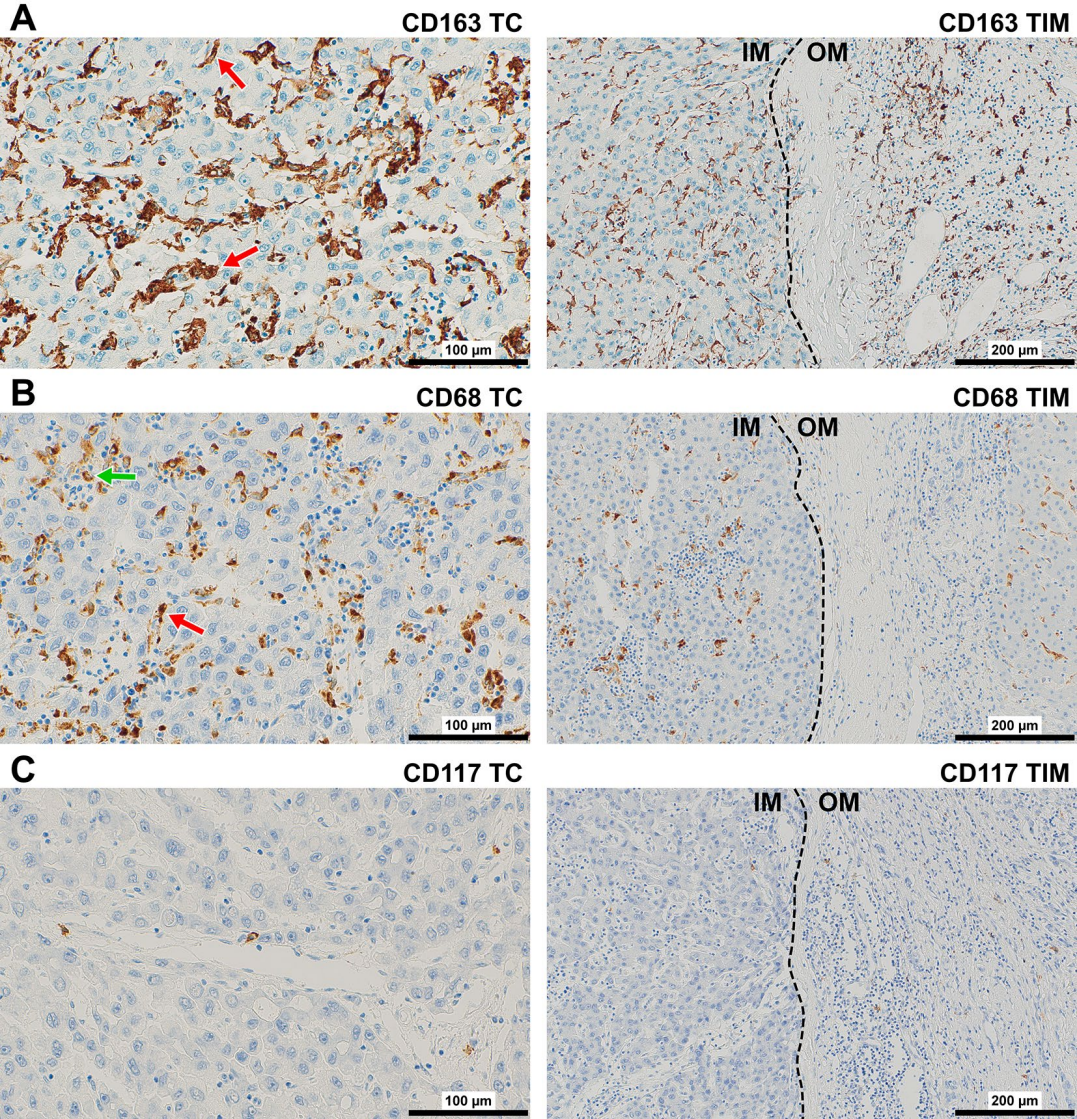
Representative immunostaining of (**A**) CD163+ and (**B**) CD68+ macrophages, and (**C**) CD117+ mast cells in TC (400×) and TIM (200×) of HCC. The red arrows point to the adjoined or partially attached macrophages to the boundaries of sinusoidal like spaces. The green arrow points to CD68+ macrophage in the lumen of sinusoid-like space. The dashed line shows the border between tumor (on the left) and non-tumor tissue (on the right). Abbreviations: HCC, hepatocellular carcinoma; TC, tumor center; TIM, tumor invasive margin, IM, inner margin (on the left) and OM, outer margin (on the right)

CD163-protein was observed intracellularly and in the cytoplasmic membrane of elongated or stellate-shaped macrophages throughout all regions of interest (ROI) (Fig. 1A). In TC and IM, they adjoined or were partially attached to the boundaries of sinusoid-like spaces or enwrapped tumor cells with few cells scattered in the stroma. In OM and PT area, CD163+ cells looked smaller and were observed in the tumor capsule, inside lymphatics, along sinusoids as well as in the stroma of portal spaces.

CD68-protein was displayed in cytoplasmic granules of rounded or elongated macrophages in all ROI (Fig. 1B). In TC and IM, they were found in the lumen and attached to the boundaries of sinusoid-like spaces or inside acini, but tumor-associated stroma harboured higher numbers of positive cells. In OM and PT area, CD68+ macrophages were observed at low frequencies in the tumor capsule as well as along the boundaries of sinusoids and in the stroma of portal spaces. Most macrophages in all ROI expressed both markers, whereas a minor part of macrophages expressed only CD68 or CD163 (Additional file 1: Fig. S2). The ratio “AF of CD163” / “AF of CD68” was greater in OM (mean 11.4, 95%CI: 0.4–30.8) and PT area (mean 10.1, 95%CI: 0.5–32.5) compared with IM (mean 7.3, 95%CI: 0.8–14.5) or TC (mean 4.7, 95%CI: 0.7–15.1), although only difference between PT area and TC was significant (*P* = 0.040).

CD117-protein was found preferentially in cytoplasmic membrane of rounded cells (Fig. 1C). In the TC and IM, mast cells were mostly localized in the stroma and in the perivascular spaces. In OM and PT area, mast cells were observed in the tumor capsule and in the perivascular spaces.

**Fig. 2:**
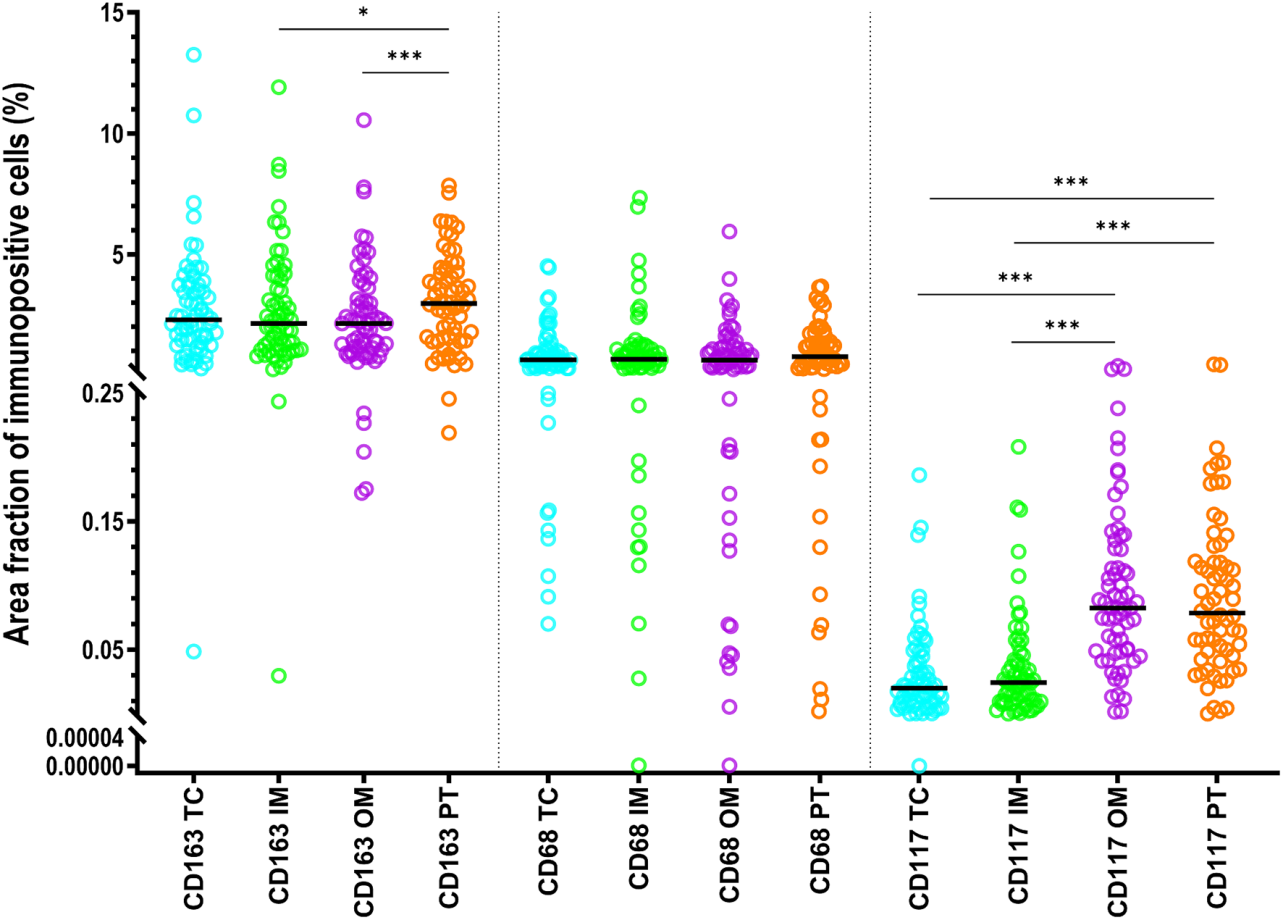
Statistics depicting the area fraction of CD68+ and CD163+ macrophages, and CD117+ mast cells in the TC, IM, OM, and PT area of HCC. Black lines are medians. Abbreviations: HCC, hepatocellular carcinoma; TC, tumor center; IM, inner margin; OM, outer margin; PT, peritumor area. *: *P* < 0.05, ***: *P* < 0.001

CD163+ and CD68+ macrophage populations displayed a significantly greater AF than CD117+ mast cells in each ROI (*p* < 0.001), at that AF of CD163+ macrophages was significantly greater than AF of CD68+ macrophages (Fig. 2, Additional file 1: Fig. S2). AF of CD163+ macrophages in IM or OM was significantly smaller than in PT area (*P* < 0.05). AF of CD117+ mast cells in TC or IM was significantly smaller than those in the PT area and OM (*p* < 0.001) (Fig. 2). AF for CD68+ macrophages did not differ significantly between regions.

Significant association for individual immune cells between all ROI are shown in: Table S5 (Additional file 1). Fig. S3 (Additional file 1) shows the heat map for the significant association between different immune cells within different regions. AF of CD68+ and CD163+ macrophages strongly correlated in all ROI. AF of CD117+ mast cells significantly correlated with that of CD68+ macrophages in TC, OM and PT area.

### Association between subtypes of immune cells and clinical and pathological variables

Tables S6–S7 (Additional file 1) shows correlations of AF of immune cells with clinical and pathological variables. High AF of all immune cells in TC correlated positively with the amount of stromal component, which confirm their stromal localization. High AF of CD68+ and CD163+ macrophages in OM and PT were associated positively with presence of microsatellites and the invasive growth patterns.

### Prognostic value of clinical and pathology variables

Among clinical and pathology variables younger age only was associated with a higher risk of recurrence, whereas no variable was associated with DFS and OS (Additional file 1: Table S8). As for non-viral cohort, both age and amount of stroma inside the tumor were significantly associated with DFS and TTR (Additional file 1: Table S9).

### Prognostic value of a single type of immune cells

**Fig. 3:**
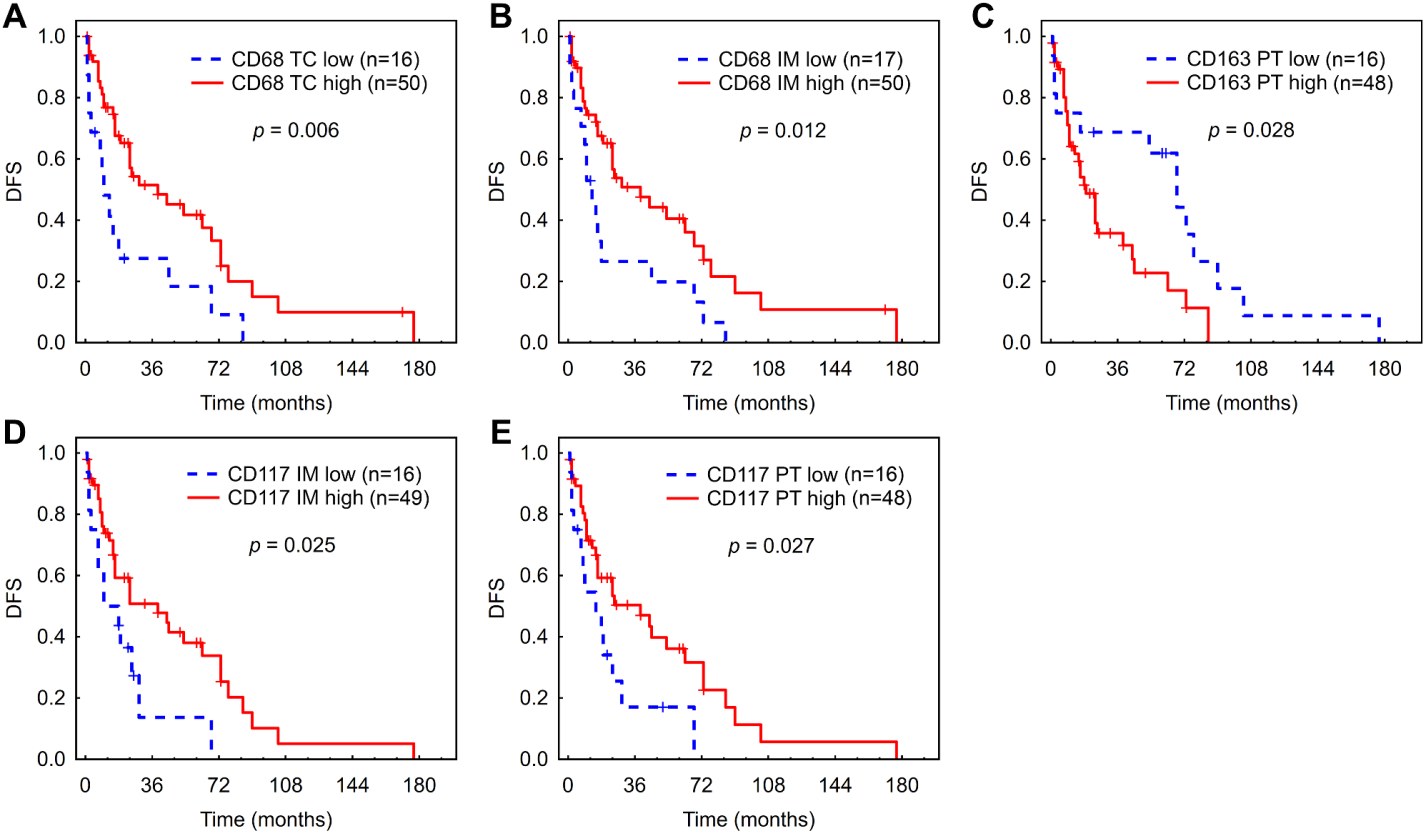
Kaplan-Meier analysis for DFS according to low vs. high AF of macrophages and mast cells in the tumor center (**A**), inner margin (**B**, **D**) and peritumor area (**C**, **E**) of HCC. (**A**), (**B**) - CD68+ macrophages. (**C**) - CD163+ macrophages. (**D**, **E**) - CD117+ mast cells. Abbreviations: DFS, disease-free survival; HCC, hepatocellular carcinoma; TC, tumor center; IM, inner margin; PT, peritumor area; n, number of patients at risk

High AF of CD68+ macrophages in TC and IM was associated with longer DFS in Kaplan-Meier analysis (Fig. 3A, B). Patients with high AF of CD163+ macrophages in the peritumor area had a significantly shorter DFS (Fig. 3C). High AF of CD117+ mast cells in IM and PT area was associated with longer DFS (Fig. 3D, E). The aforementioned results were confirmed also in Cox regression (Additional file 1: Fig. S4). The associations between the AF of immune cells in each ROI and DFS for non-viral cohort (Additional file 1: Table S10) were very close to those obtained for the whole cohort in terms of HRs and p-values (Additional file 1: Fig. S4).

Prognostic values of analyzed immune cells for TTR and OS revealed lower number of significant associations; however, general pattern was similar to that for DFS (see Additional file 1: Figs. S4–S9, Table S10).

To eliminate the potential confounding effect of systemic treatment due to recurrence we excluded 13 respective patients and performed additional Cox regression for DFS and OS (Additional file 1: Table S11). All associations between AFs of immune cells and survival, which were significant in the whole cohort, remained.

### Multivariate analysis

We tested the associations of immune cells, which had significant associations with DFS in the non-viral cohort in a multivariable model after adjustment for age and proportion of stromal component. For all models, both age and proportion of stromal components were significantly associated with DFS. Only AF of CD117+ mast cells in IM retained a significant association with DFS (Additional file 1: Table S12).

### Prognostic value of combinations of immune cells

#### CD68+ and CD163+ macrophages

**Fig. 4:**
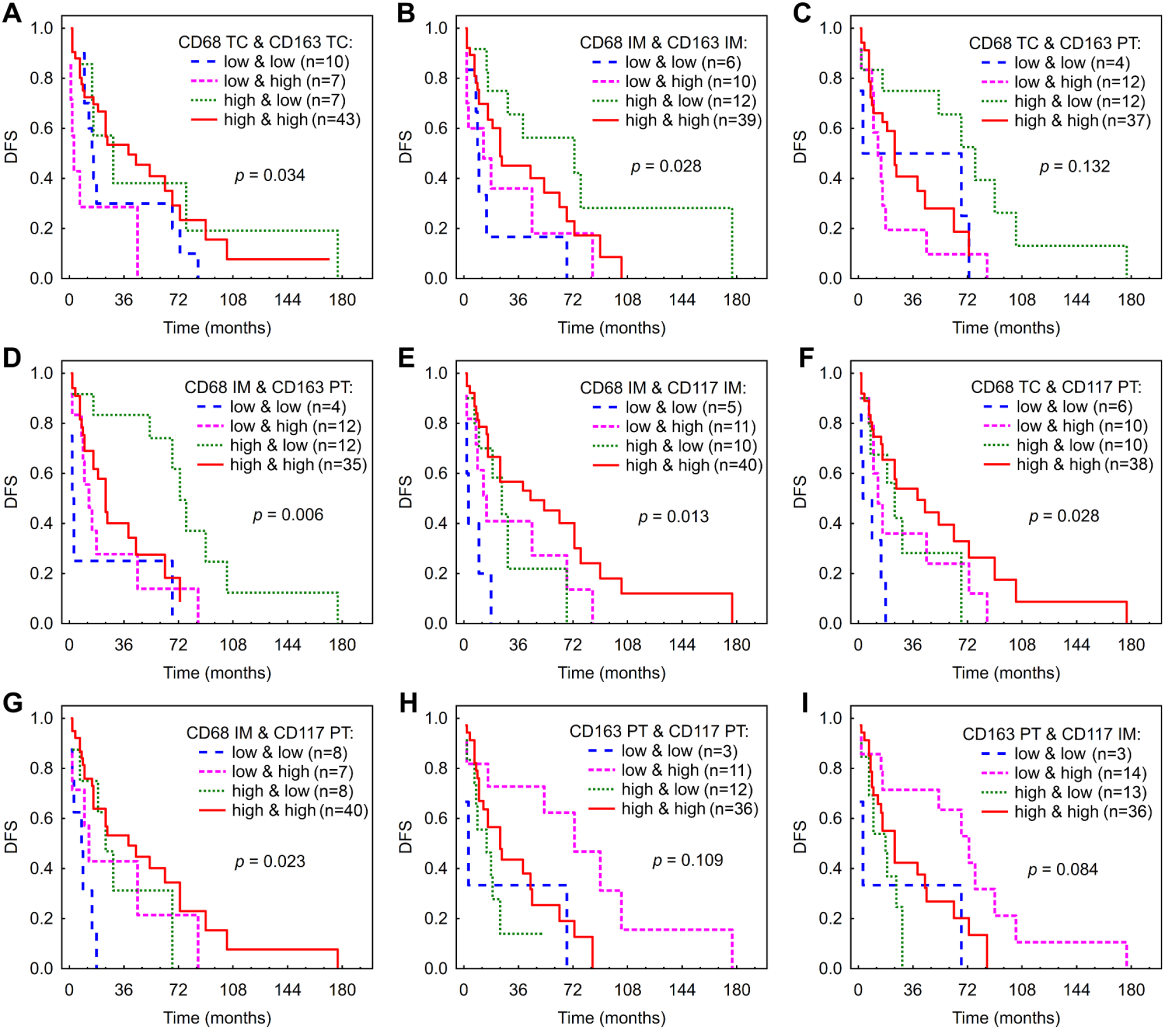
Kaplan-Meier analysis for DFS according to combined low vs. high AF of macrophages and mast cells in different regions of HCC. (**A**) - CD68 TC & CD163 TC. (**B**) - CD68 IM & CD163 IM. (**C**) - CD68 TC & CD163 PT. (**D**) - CD68 IM & CD163 PT. (**E**) - CD68 IM & CD117 IM. **F **- CD68 TC & CD117 PT. (**G**) - CD68 IM & CD117 PT. (**H**) -CD163 PT & CD117 PT. (**I**) - CD163 PT & CD117 IM. Abbreviations: DFS, disease-free survival; HCC, hepatocellular carcinoma; TC, tumor center; IM, inner margin; PT, peritumor area; n, number of patients at risk

In order to highlight an aggregate effect of CD68- and CD163-expressing macrophages, we categorized patients according to their AFs of those markers into high/high, low/low, high/low and low/high groups. Patients from CD68TC^low^ & CD163TC^high^ group displayed significantly shorter DFS compared to all the rest of the groups (*P* < 0.05) (Fig. 4A). As for IM, group of CD68IM^high^ & CD163IM^low^ demonstrated longer DFS compared to low AF of both markers in Kaplan-Meier (*P* = 0.004) (Fig. 4B) and in Cox regression (Additional file 1: Fig. S5, Table S13). To investigate whether the combined analysis of two regions could improve the prediction of patient survival, we combine AFs of CD68+ and CD163+ macrophages in two different regions. Patients from CD68TC^high^ & CD163PT^low^ group had longer DFS compared to CD68TC^low^ & CD163PT^high^ and to CD68TC^high^ & CD163PT^high^ (*P* = 0.005, 0.031, respectively (Fig. 4C). Patients from CD68IM^high^ & CD163PT^low^ group demonstrated longer DFS compared to all the rest of the groups in Kaplan-Meier analysis (*p* < 0.008) (Fig. 4D), and showed the association with DFS in Cox regression (Additional file 1: Fig. S5, Table S13).

### CD68+ macrophages and CD117+ mast cells

For IM, patients with high AF of both markers and only CD68^high^ had significantly longer DFS compared to those with low AF of both markers (Fig. 4E, Additional file 1: Fig. S5, Table S13).

Patients with CD68TC^high^ & CD117PT^low^ and CD68TC^high^ & CD117PT^high^ had significantly longer DFS compared to low/low group (*p* = 0.012 and 0.001, respectively) (Fig. 4F). Cox regression showed association of CD68TC^low^ & CD117PT^high^, CD68TC^high^ & CD117PT^low^ and CD68TC^high^ & CD117PT^high^ groups with DFS (Additional file 1: Fig. S5, Table S13).

CD68IM^high^ & CD117PT^low^ and CD68IM^high^ & CD117PT^high^ groups had significantly longer DFS compared to low/low group (*p* = 0.010 and 0.001, respectively) (Fig. 4G), which was confirmed in Cox regression (Additional file 1: Fig. S5, Table S13).

### CD163+ macrophages and CD117+ mast cells

Patients from CD163PT^low^ & CD117PT^high^ group had significantly longer DFS compared to all the rest of the groups (*p* < 0.05) (Fig. 4H). In Cox regression, CD163PT^low^ & CD117PT^high^ group had a significant association with DFS (Additional file 1: Fig. S5, Table S13).

CD163PT^low^ & CD117IM^high^ group had significantly longer DFS compared to all the rest of the groups in Kaplan-Meier analysis (Fig. 4I) and compared to low/low group in Cox regression (Additional file 1: Fig. S5, Table S13).

## Discussion

In our study, we analysed the TME of HCC after a curative resection in terms of type, density, location and eventual interactions of macrophages and mast cells within distinct tumor regions. High AF of CD68+ macrophages in TC and IM were associated with a lower risk of recurrence and a longer DFS. High AF of CD163+ macrophages in PT area correlated with a shorter DFS. High AF of mast cells in IM and PT area was associated with a longer DFS. When we looked at an aggregate impact of different cell types, the best outcomes were found for patients with high AF of antitumor CD68+ macrophages and CD117+ mast cells along with low AF of protumor CD163+ macrophages. Accordingly, patients with CD163TC^high^ along with CD68TC^low^ had the shortest DFS, and cases with CD163IM^low^ along with CD68IM^high^ demonstrated significantly longer DFS compared to low AF of both markers. The longest DFS was also characteristic for CD68IM^high^ & CD163PT^low^, CD117IM^high^ & CD163PT^low^ and CD117PT^high^ & CD163PT^low^ groups.

### CD68+ and CD163+ macrophages

We assessed the prognostic role of all tumor-associated macrophages, which express CD68, and their pro-tumor M2 subset, expressing CD163 [33] individually and in combination. The ratio between CD68- and CD163-expressing macrophages was used earlier to assess the polarization of TAMs and enhance the accuracy of their prognostic impact in HCC [9, 11].

Most macrophages in all ROI expressed both markers, whereas a minor part of macrophages expressed only CD68 or CD163. Only CD68+ macrophages had a rounded appearance (in agreement with [34]) (see Fig. 1). Higher AF of CD163+ macrophages in all ROI along with the spindle-like shape of the majority of cells implies that most macrophages in HCC microenvironment were polarized into M2 or M2-like phenotype, which is in agreement with the literature [11, 16, 35]. During the process of polarization to M2-type, the expression of CD68 is downregulated and the expression of CD163 is upregulated. Another possible explanation for the higher area fraction of CD163+ macrophages is that CD163 was expressed on the cell surface and in the intracellular compartment in our and earlier studies [36], whereas CD68 in our study was displayed only in cytoplasmic granules as a lysosomal membranes-associated protein [37]. In addition, some dendritic cells may express CD163 [38]. Higher AF of CD163 vs. CD68 agrees with the observations in HCC [11], liver metastasis of colorectal cancer [39], and breast cancer [40].

Survival analysis shows that TAM expressing CD68 displayed antitumor properties only in the TC and IM. Higher expression of CD68 along with its lysosomal localization may in turn imply enhanced phagocytosis of tumor cells [41]. In addition, an indirect killing of tumor cells through antibody-dependent cellular cytotoxicity and direct killing through the release of harmful products by TAM cannot be overlooked [42, 43]. This also holds for the role of TAM in the processing and presentation of cancer antigens to CD4+ T cells and the engagement of adaptive anticancer immunity [44]. Several previous studies on HCC and other cancers reported results concordant to ours for tumor invasive front [42, 45] and for tumor core [46, 47].

In our cohort, the expression of CD163 was associated with a clear protumor action only in PT area. The highest expression of CD163 in PT area may reflect predominance of protumor M2-polarized macrophages in this region. AF of CD163+ TAM in the PT region significantly correlated with invasive growth patterns of the HCC and with presence of microsatellites. These findings may indicate that the invasion of tumor cells into PT area is responsible for the activation of Kupffer cells and their polarization into M2 [48] along with the polarization of M1 or naïve macrophages into the M2 type. The ability of macrophage-colony stimulating factor produced by tumor cells [49], to induce the macrophage polarization into M2 type has been demonstrated recently [50].

Protumor influence of CD163+ TAM may be a consequence of their involvement into enhanced tumor cell invasion [51], proliferation, angiogenesis, and metastasis [52] along with inhibition of antitumor T-cell responses [53]. In parallel, CD163+ macrophages may prevent activation of M1-macrophages [41]. Earlier studies have also shown the association of high PT CD163+ M2-TAMs with worse clinical prognosis in HCC [54, 55], although the ROI were not well defined.

When we analysed the impact of combined expression of both markers on survival, we reached the conclusion that the ultimate effect of TAM in HCC TME depends on the fraction of putative protumor CD163+ macrophages among the whole macrophage population within particular region as well as in the whole tumor TME. Indeed, high expression of CD68 in the IM along with low expression of CD163 in the PT region was significantly associated with the longest DFS, highlighting the most favourable outcome. Moreover, CD68^low^ & CD163^high^ expression in the TC was associated with the shortest DFS compared to all other possible combinations between CD68+ and CD163+, and CD68^high^ & CD163^low^ expression in the IM was associated with longer DFS. Therefore, macrophages with only or predominant CD163 expression exert protumor action in the tumor as well, however, this effect is counterbalanced by expression of CD68. In fact, the ratio CD163/CD68 in our cohort was greater in PT area compared with TC. These observations can mechanistically explain why the antitumor effects of CD68-expressing macrophages predominate in the TC and IM, and the protumor effects of CD163+ macrophages are evident in PT area. Earlier, Atanasov et al. on non-viral European population did not observe associations between the expressions of CD163 in the TC or tumor invasive margin with survival either [45]. It seems that complex associations between several phenotypes should be taken into account for informed judgment regarding significance of tumor-infiltrating immune cells. Our hypothesis is in line with recent concepts [56] according to which macrophages in liver can be divided into resident Kupffer cells (CD68+ CD163+) of embryonic origin and blood/bone marrow-derived macrophages (CD68+ CD163-). Both types of macrophages can regulate their functional phenotype according to the signals from the HCC microenvironment [52]. In HCC, which associates with secretion of chemokines and growth factors by the stromal cells and tumor cells, monocyte-derived macrophages can acquire a phenotype of Kupffer cells [35, 52] and may start expression of putative markers, including CD163 [53]. Furthermore, monocyte-derived macrophages can polarize within HCC into antitumor, proinflammatory M1 (CD68+ CD163-) and protumor, antiinflammatory M2 (CD163+) macrophages [57]. This concept explains why most macrophages in the HCC microenvironment were polarized to full M2 or M2-like-phenotype [52, 58] in ours and other studies [59] as well as clarifies the frequent colocalization of CD68 and CD163, which may mirror the transition of naive and M1 macrophages into M2 phenotype. Since macrophage function is linked to their phenotype and location, one needs to consider the macrophages’ functional anatomical classification when interpreting their interactions with tumors.

### CD117+ mast cells

Longer DFS in patients with high AF of CD117 in the IM and PT area suggests a survival advantage conferred by CD117+ mast cells. The influx of mast cells into TME is mediated by tumor-cell-released chemoattractants, such as SCF or CCL15, and then mast cells can actively participate in the elimination of tumor cells through secreted histamin, TNF-α, and IL-1, IL-4 and IL-6 [19]. Positive correlation between AF of mast-cells and CD68+ macrophages inside and between regions (CD68+ cells in TC and CD117+ cells in IM) supports the findings that mast cells may also promote the attraction of monocytes into the tumor [21]. Although survival advantage was conferred by both mast cells and CD68+ macrophages, we were not able to prove cooperation between them, since CD68IM^high^ & CD117IM^high^, CD68TC^high^ & CD117PT^high^ and CD68IM^high^ & CD117PT^high^ groups did not have survival advantage compared to only CD68^high^ or CD117^high^ groups. Small sample size can be a reason for this; nevertheless, both cell types operate as independent antitumor effectors, as such CD68+ macrophage effects dominated in the TC and IM, whereas CD117+ mast cells effects were more obvious in IM and PT area.

A comprehensive investigation of a larger cohort of 245 HCC patients found a positive association between greater mast cells infiltration in tumor samples and longer survival after tumor resection [8]. Giuşcă et al. showed that PT CD117+ mast cells in patients with liver metastases of colorectal origin were significantly correlated with longer overall survival [60]. As in our study, Rohr-Udilova et al. reported greater densities of mast cells in surrounding HCC tissue, however, only intratumor mast cells density was associated with lower recurrence rate [5].

The detected antitumor properties of mast cells and protumor activity of CD163+ macrophages were strengthened by vanishing of positive effect of mast cells in IM on DFS and TTR in patients with concomitant high AF of CD163+ cells. Moreover, the longest DFS was observed in patients from CD117PT^high^ & CD163PT^low^ group and CD117IM^high^ & CD163PT^low^ group, which emphasize the importance of their intra- and inter-region communication. Our results highlight an antagonistic interaction between mast cells and CD163+ macrophages in IM and PT region and argue for the utility of complex assessment of TME, which may include several phenotypes of immune cells [2]. The significant antitumor impact of CD117+ mast cells in IM and PT area was not visible in TC and OM. Along with the observed associations of CD68+ macrophages in TC and IM and CD163+ in PT region with survival, these findings underscore the importance of rigorous annotation of IM and PT area, which is not standard yet. As for absence of prognostic impact of any cell type in the OM, 95% of our cohort had complete or partial capsule around the tumor, which, indeed, overlapped with the OM region. High amount of connective tissue may complicate the communication between immune cells in OM. Most of stromal elements collaborate to form an immunosuppressive TME that enables cancer cells to escape immune surveillance [61].

Altogether, the associations of proinflammatory mast cells and CD68+ macrophages with better outcomes after HCC resection, along with antagonistic effect of antiinflammatory CD163+ macrophages, indicate the importance of tumor-associated inflammation as a major mechanism of antitumor immunity [19]. Despite the triggering role of inflammation in tumor development [9, 62], it may serve as a protective barrier against tumor growth and metastasis [19]. Further research is needed to characterize mast cells in HCC microenvironment and their role in the pathology of HCC.

Contrary to our results, intratumoral, marginal, or peritumoral abundance of CD68+ TAMs in HCC was associated negatively with survival in some studies [11, 25]. Higher peritumoral and intratumoral mast cells were associated with poorer clinical outcomes in resected HCC patients in two studies [6, 7]. However, a vast majority of patients in those studies had hepatitis B-associated HCC, which could be a reason for the protumor action of macrophages [63]; in addition, ROI was defined differently and only selected fields of view were analysed.

In our earlier paper on the same cohort, we demonstrated prognostic associations of T-cells and B-cells in the TME of HCC [2]. It will be an interesting question to search for interactions of innate and adaptive immune cell interactions on the risk and outcome of HCC. This will be a full study of its own but our preliminary results suggest that such interactions may be found (https://www.livercancer.de/conference/abstractbook/ pages 41–42).

### Findings from a non-viral cohort

After the exclusion of viral-induced cases, most significant associations between immune cells and outcomes were preserved, so associations between macrophages and mast cells and prognosis after resection of HCC might be extrapolated to the population of non-viral-induced HCC. Recently published reports suggest that the efficacy of immunotherapy can differ between viral- and in non-viral-induced HCC [64], and finding predictive and/or prognostic biomarkers for both cohorts is very important. We believe that our findings (mainly based on non-viral HCC) could help to identify patients at risk, who could be candidates for adjuvant immunotherapy based on immune checkpoint inhibitors or various novel strategies, e.g., targeting tumor-associated macrophages or mast cells (reviewed in [65, 66]), what can be tested in future studies.

Moreover, we consider of interest observed associations between the higher amount of stroma inside the tumor with longer TTR and DFS in the non-viral cohort, and mechanisms of such impact are awaiting investigation. The stromal compartment harbours the majority of tumor-infiltrating immune cells in different cancers, HCC included [2, 67, 68] and indeed, stroma-infiltrating lymphocytes showed better associations with outcomes [69, 70]. Tumor-stroma-based therapeutic targeting [61] showed promise in enhancing innate and adaptive antitumor immunity.

#### Limitations

The current study has several limitations, including its retrospective nature, in addition to the relatively small number of patients and the absence of a validation cohort.

None of the patients included in our study were treated with immune checkpoint inhibitors in the adjuvant or palliative setting. Thus, we cannot conclude the predictive role of macrophages and mast cells for immune checkpoint inhibitors-based therapy, however, our findings may promote future research or clinical trials.

The low prevalence of hepatitis and cirrhosis hamper the comparability of our results to other cohorts, particularly of Asian patients. On the other hand, our study provides insight into the TME of HCC in patients with non-viral etiology of the disease. Considering the complexity of macrophage population as well as the interactions between different immune cells, more IHC markers and multiplex staining are needed to obtain reliable picture of the immune TME of HCC, which is the scope of our ongoing researches.

## Conclusion

Our study on HCC patients after resection highlighted the survival advantage in the case of high AF of CD68+ macrophages in TC and IM and high AF of CD117+ mast cells in IM or PT area, along with low AF of CD163+ macrophages in PT area. The ultimate effect of macrophages and mast cells is context-dependent, i.e. modified by the abundance of other cell phenotypes in TME. Our results also pointed out the importance of standardized annotation of ROI including individual assessment of IM and OM, as well as the importance of the PT region, which is frequently overlooked. The results offer a new approach to stratify patients after curative liver resection and identify those at risk of recurrence and death.

### Electronic supplementary material

Below is the link to the electronic supplementary material.

Supplementary Material 1

## Data Availability

All data generated or analyzed during this study are included in this article and its additional material files. Further enquiries can be directed to the corresponding author.

## Abbreviations

HCC: Hepatocellular carcinoma
AF: Area fraction
TC: Tumor center
IM: Inner margin
OM: Outer margin
PT: Peritumor area
TTR: Time to recurrence
DFS: Disease-free survival
OS: Overall survival
TME: Tumor microenvironment
TNM: Tumor-node-metastasis
TAM: Tumor-associated macrophages
FDR: False discovery rate
NASH: Non-alcoholic steatohepatitis
ROI: Regions of interest
HR: Hazard ratio
NAFLD: Non-alcoholic fatty liver disease
RFP: Recurrence-free proportion
CI: Confidence interval
