# Supplementary material for: Prognostic role of macrophages and mast cells in the microenvironment of hepatocellular carcinoma after resection
Source: BMC Cancer. 2024 Jan 29;24:142. doi: 10.1186/s12885-024-11904-8 (PMC10823625; doi:10.1186/s12885-024-11904-8)
Supplement: Supplementary file 1 — Supplementary Material 1 [file 12885_2024_11904_MOESM1_ESM.docx]

**Supplementary data**

**Fig. S1**: Screen shoot of the QuPath software window


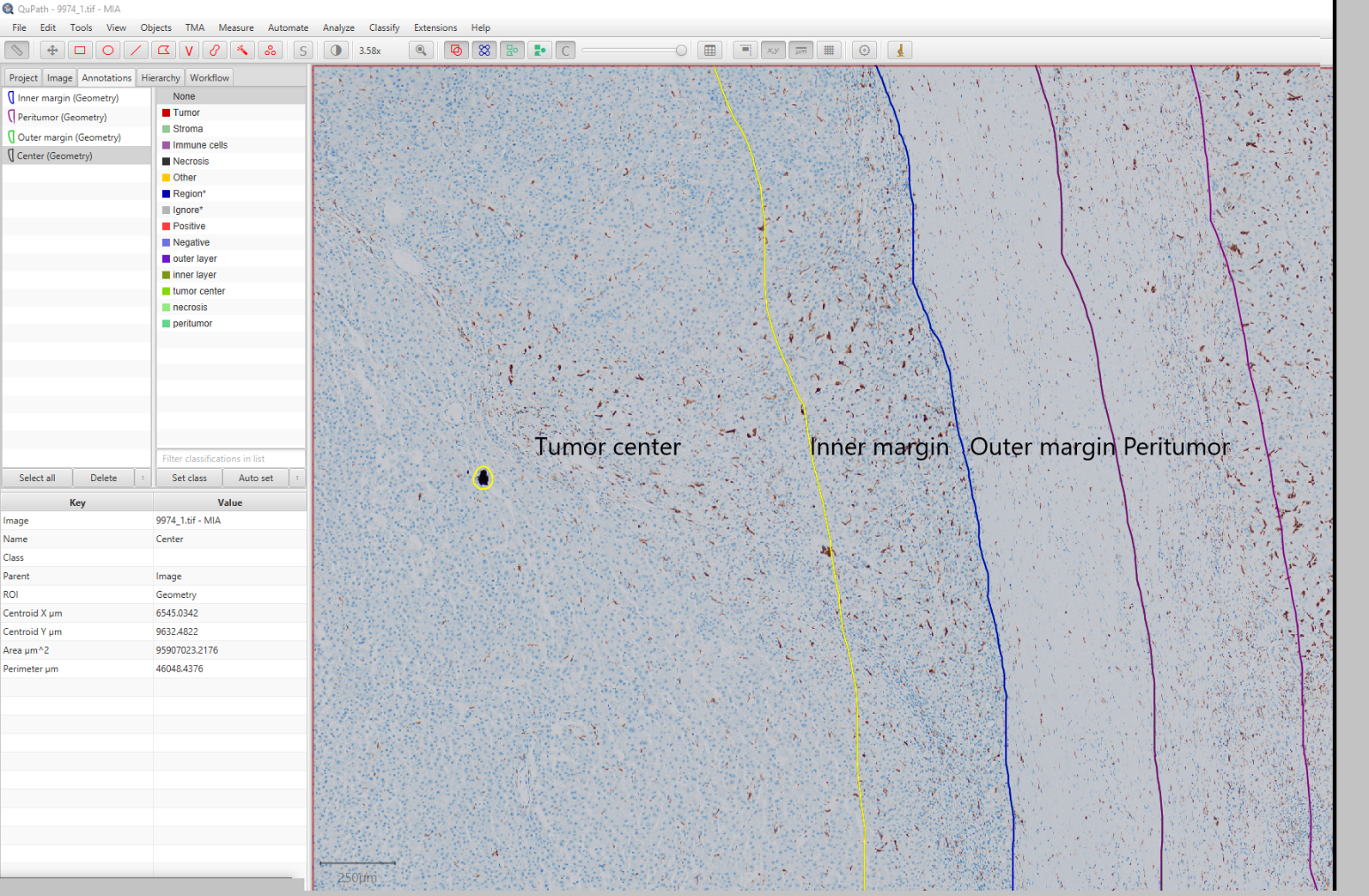


This screen shoot shows automaticly annotated regions of interest (ROIs) in hepatocellular carcinoma using QuPath: tumor center, inner margin, outer margin, and peritumor area. Scale bar 250 µm.

**Fig. S2**: Representative Immunostaining of macrophages in HCC tissue


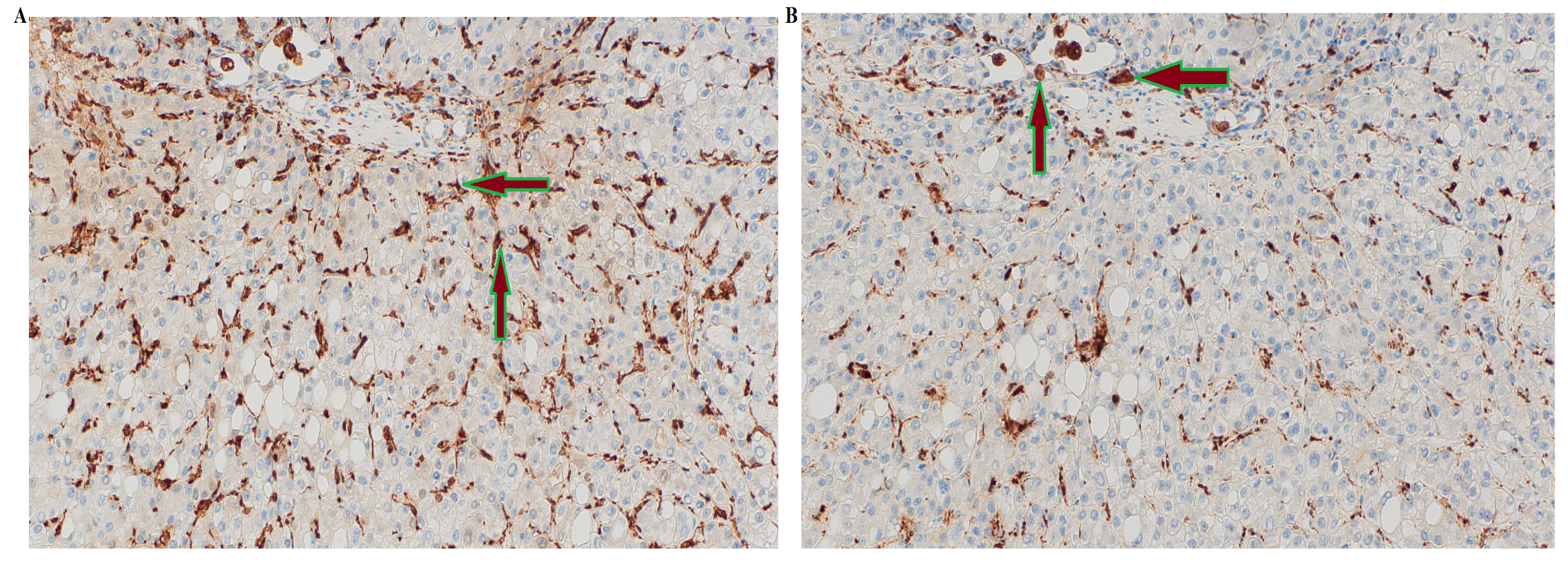


1. CD163+ cells in the tumor center of hepatocellular carcinoma, 400×. Arrows: only CD163+ only positive cells. B. CD68+ cells in the tumor center of hepatocellular carcinoma, 400×. Arrows: only CD68+ positive cells. Most macrophages express both markers.

Abbreviations: HCC, hepatocellular carcinoma

**Fig S3:** Heat map for significant correlations between area fraction of macrophages and mast cells in different regions of interest


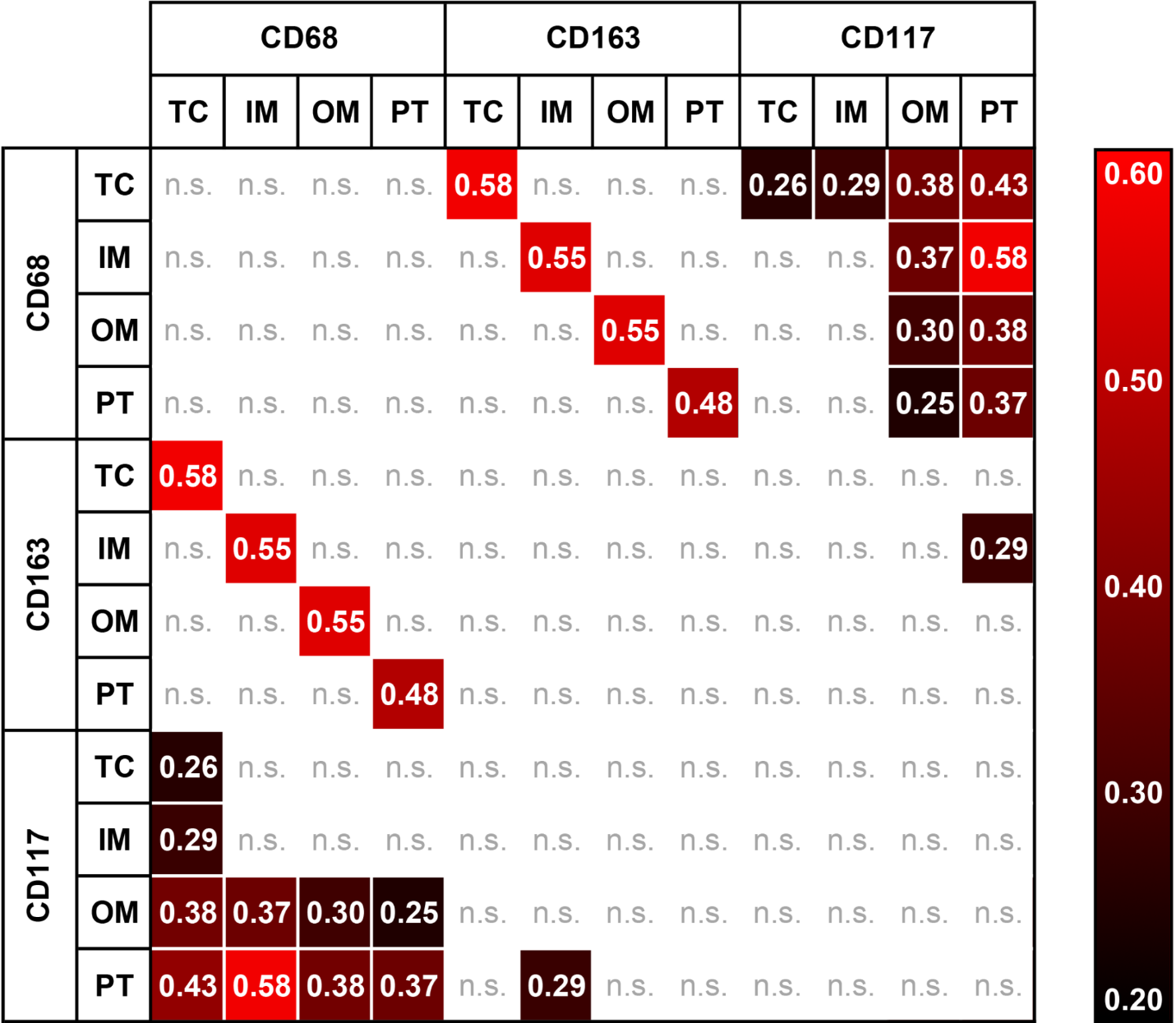


Heat map for significant correlations between area fraction of CD68+ and CD163+ macrophages and CD117+ mast cells in TC, IM, OM, and PT (Spearman ρ, P<0.05).

Abbreviations: TC, tumor center; IM, inner margin; OM, outer margin; PT, peritumor area

**Fig. 4** Heat map for the univariable survival analysis of the association of area fraction of macrophages and mast cells


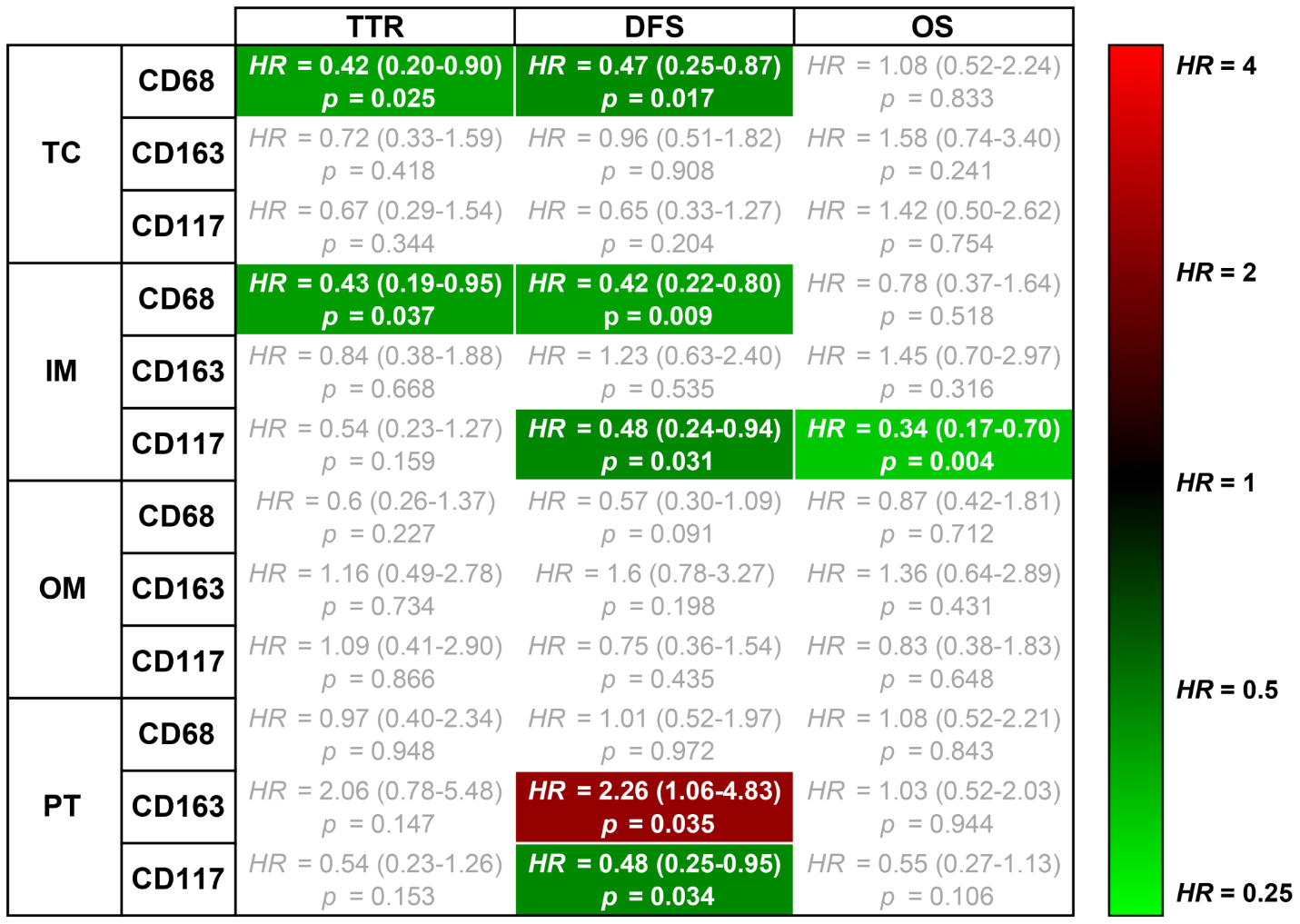


Heat map for the univariable analysis of the association of area fraction of CD68+ and CD163+ macrophages and CD117+ mast cells with TTR, DFS, and OS per individual region of interest using the Cox regression (67 HCC patients). Significant results are color-graded. For all cells and regions of interest, the area fraction of immune cells per area section (mm^2^) was converted into percentiles and then categorized into low (0-25 percentile) vs high (25-100 percentile). Hazard ratios show the relative risk compared with 1 for the low AF.

Abbreviations: HR, hazard ratio; TTR, time to recurrence; DFS, disease-free survival; HCC, hepatocellular carcinoma; OS, overall survival; TC, tumor center; IM, inner margin; OM, outer margin; PT, peritumor area

**Fig. S5**: Heat map for the univariable survival analysis of the association of combined two types of immune cells


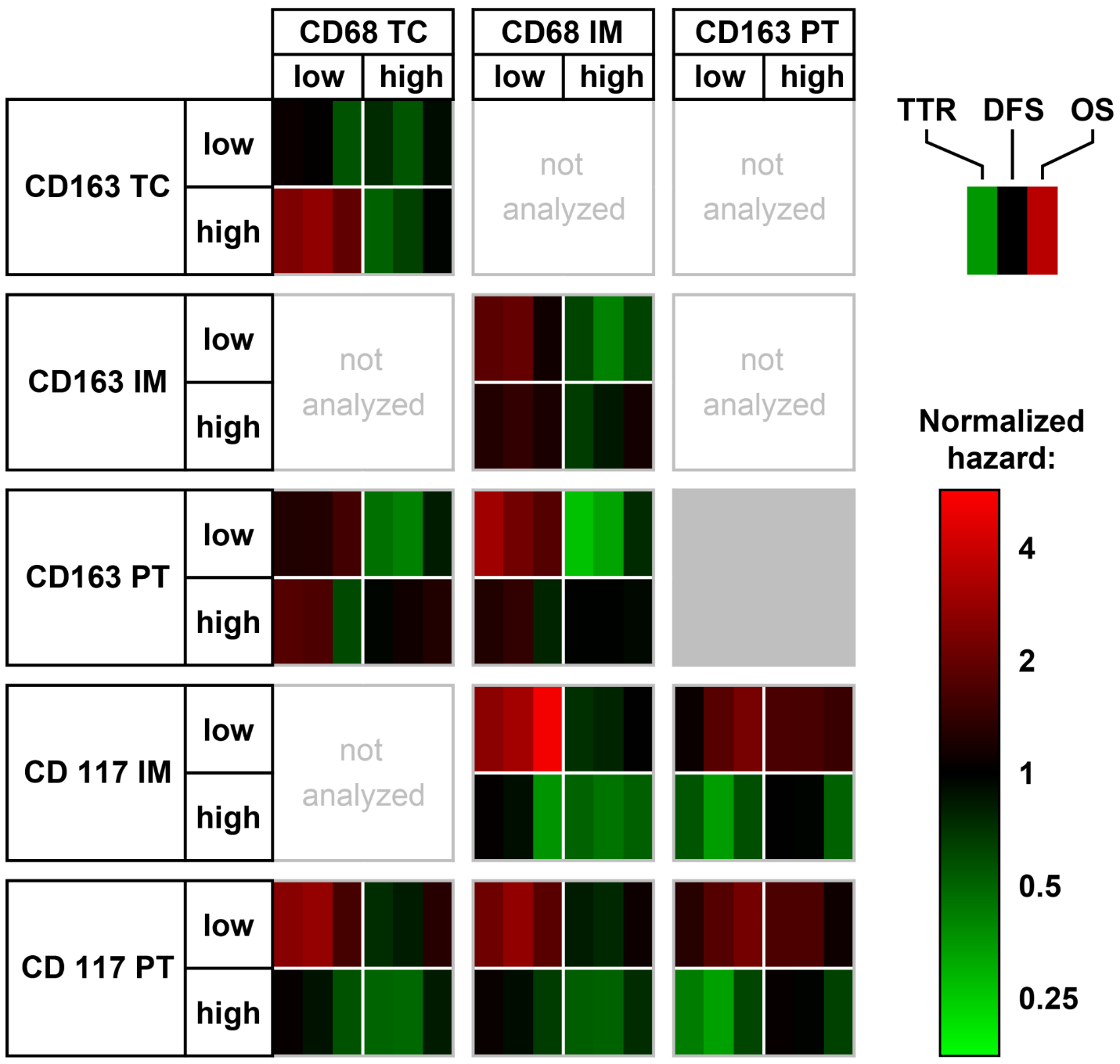


Heat map for the univariable analysis of the association of combined two types of immune cells with TTR, DFS, and OS within different regions using the Cox regression (67 HCC patients). For all cells and regions of interest, the area fraction of immune cells per area section (mm^2^) was converted into percentiles and then categorized into low (0-25 percentile) vs high (25-100 percentile). The hazard levels shown in the heat map were normalized so that their geometric mean across all four groups is equal to one (independently for TTR, DFS and OS).

Abbreviations: TTR, time to recurrence; HCC, hepatocellular carcinoma; DFS, disease-free survival; OS, overall survival; TC, tumor center; IM, inner margin; PT, peritumor area

**Fig. S6:** Kaplan–Meier TTR and OS curves for combined AF of tumor-infiltrating immune cells in HCC patients


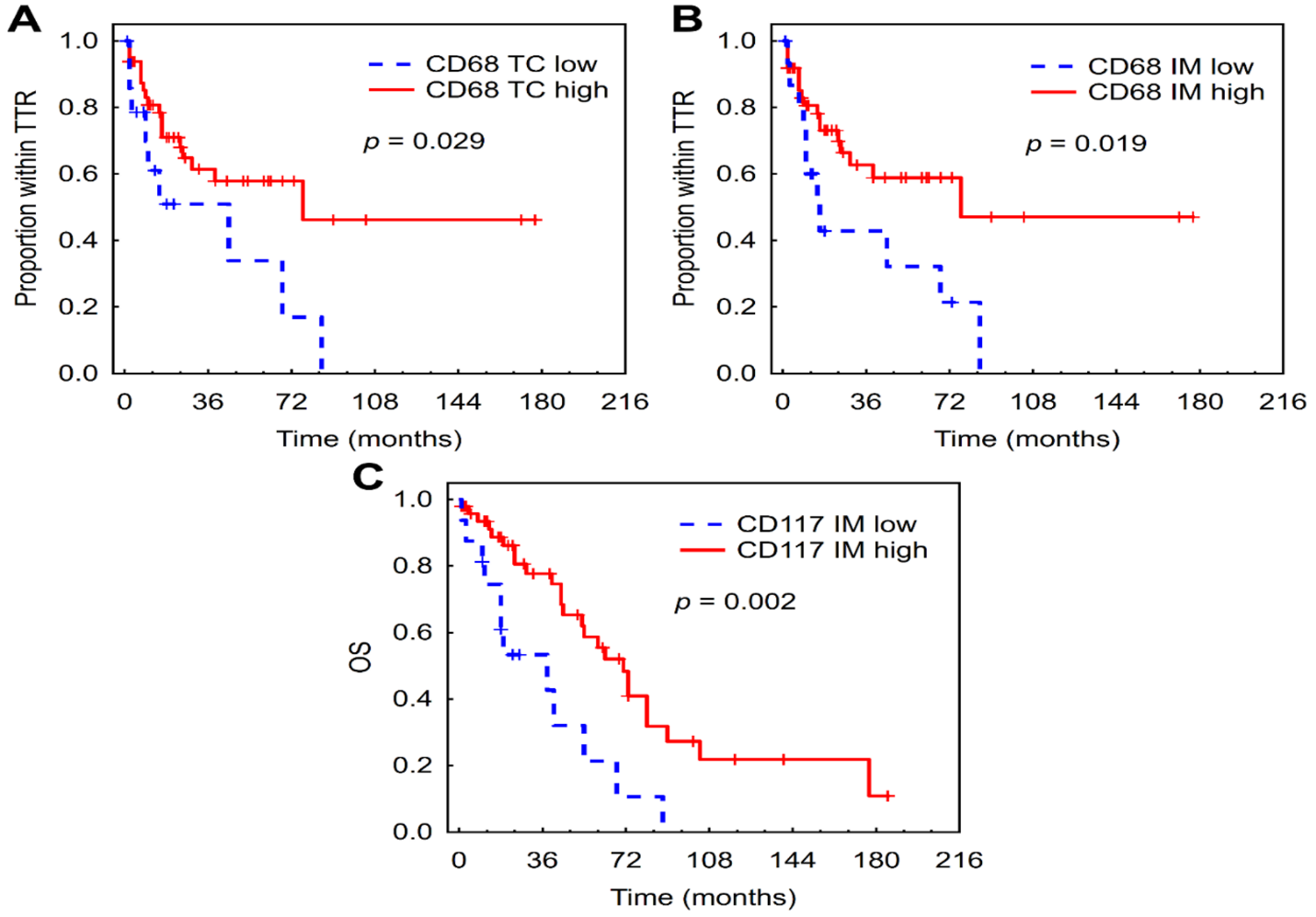


Kaplan–Meier analysis for TTR and OS according to low vs. high AF of tumor-infiltrating immune cells in the tumor center (A) and inner margin (B, C) of HCC.

A, B - CD68+ macrophages. C - CD117+ mast cells.

Abbreviations: HCC, hepatocellular carcinoma; TTR, time to recurrence; DFS, disease-free survival; TC, tumor center; IM, inner invasive

**Fig. S7:** Kaplan–Meier TTR curves for combined AF of tumor-infiltrating immune cells in HCC patients


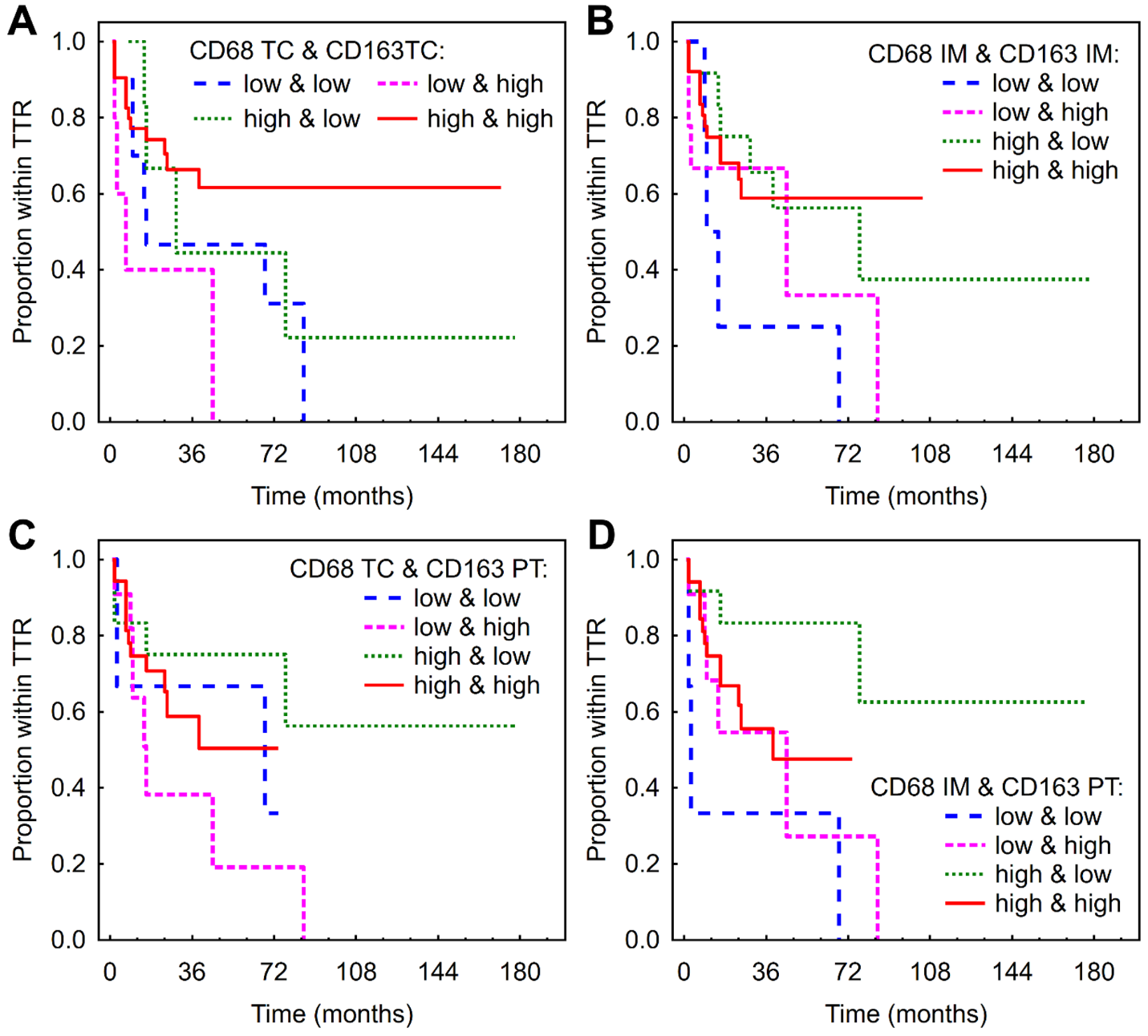


Kaplan–Meier analysis for TTR according to combined low vs. high AF of CD68+ macrophages and CD163+ macrophages in the tumor center, inner margin and peritumor area of HCC.

A - CD68 TC & CD163 TC. B - CD68 IM & CD163 IM. C - CD68 TC & CD163 PT. D - CD68 IM & CD163 PT.

Abbreviations: HCC, hepatocellular carcinoma; TTR, time to recurrence; TC, tumor center; IM, inner margin; PT, peritumor area

**Fig. S8:** Kaplan–Meier TTR and OS curves for combined AF of tumor-infiltrating immune cells in HCC patients


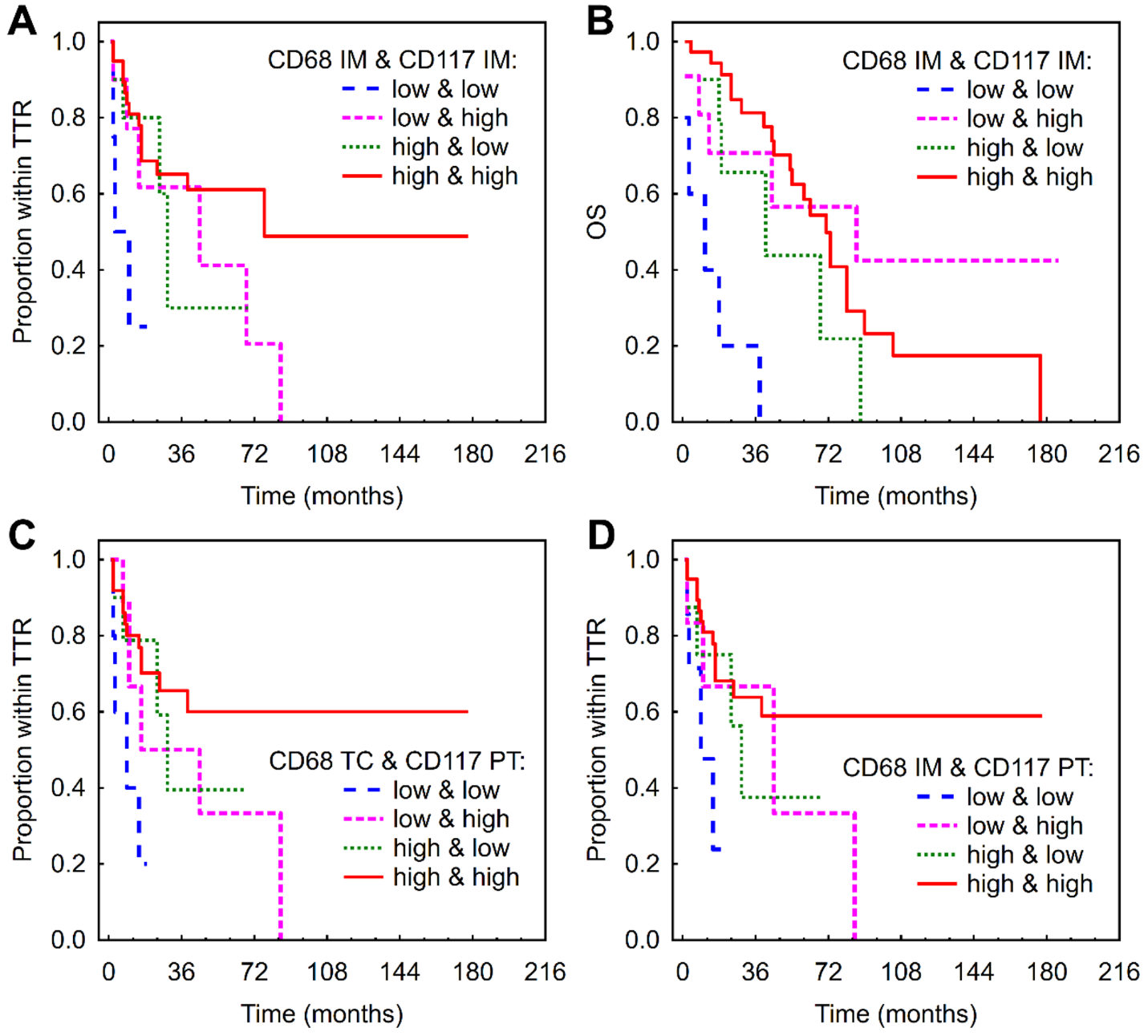


Kaplan–Meier analysis for TTR and OS according to combined low vs. high AF of CD68+ macrophages and CD117+ mast cells in the tumor center, inner margin and peritumor area of HCC.

A - CD68 IM & CD117 IM. B - CD68 IM & CD117 IM. C - CD68 TC & CD117 PT. D – CD68 IM & CD117 PT.

Abbreviations: HCC, hepatocellular carcinoma; TTR, time to recurrence; OS, overall survival; TC, tumor center; IM, inner margin; PT, peritumor area

**Fig. S9:** Kaplan–Meier OS curves for combined AF of tumor-infiltrating immune cells in HCC patients


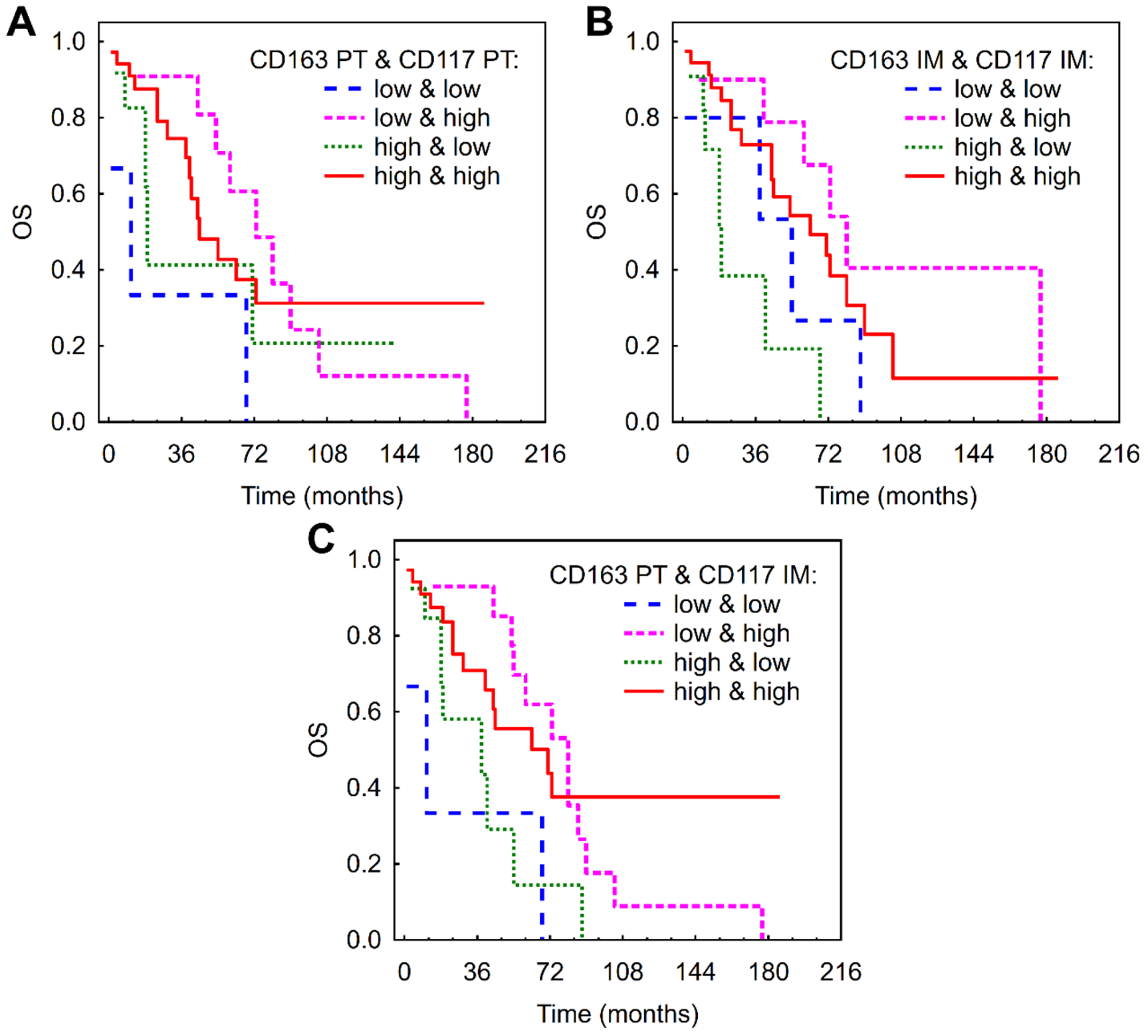


Kaplan–Meier analysis for OS according to combined low vs. high AF of CD163+ macrophages and CD117+ mast cells in the inner margin and peritumor area of HCC.

A – CD163 PT & CD117 PT. B – CD163 IM & CD117 IM. C – CD163 PT & CD117 IM.

Abbreviations: HCC, hepatocellular carcinoma; OS, overall survival; IM, inner margin; PT, peritumor area

**Table S1.** Clinical backgrounds of enrolled hepatocellular carcinoma patients

| **Variables** |  |
| --- | --- |
| No. of patients | 67 |
| Age, y (median, min-max) | 69 (24-86) |
| Sex (male/female), n (%) | 52/15 (77.6%/22.4%) |
| Background disease, n (%)  Cirrhosis  Hepatitis  Hepatitis C  Alcoholic steatohepatitis  Cryptogenic chronic hepatitis  Non-alcoholic steatohepatitis  Mixed etiology of hepatitis  NAFLD  Unknown | 15 (22.4%)  42 (62.7%)  3 (4.5%)  7 (10.4%)  7 (10.4%)  16 (23.9%)  9 (13.4%)  2 (3.0%)  8 (11.9%) |
| Diabetes Mellitus; yes/no, n (%) | 28/49 (41.8%/58.2%) |
| Metabolic syndrome; yes/no/unknown | 10/29/28 (14.9%/43.3%/41.8%) |
| Child-Pugh score, n (%)  A  B  unknown | 62 (92.5%)  3 (4.5%)  2 (3.0%) |
| Gross structure of the tumor, n (%)  single distinctly nodular  multinodular  vaguely nodular  infiltrative | 42 (62.7%)  17 (25.4%)  6 (9.0%)  1 (1.5%) |
| Tumor number; solitary/multiple, n (%) | 58/9 (86.6%/13.4%) |
| Tumor size, n (%)  ≤ 5 cm  > 5 cm  unknown | 30 (44.8%)  34 (50.7%)  3 (4.5%) |
| Tumor stage, TNM, n (%)  I  II  III  IV | 46 (68.7%)  13 (19.4%)  5 (7.5%)  3 (4.5%) |
| Alpha-fetoprotein, IU/ml (median, min-max) | 4.0 (1.0-6612.0) |
| Events, n (%)  Recurrence  Death | 29 (41.8%)  38 (56.7%) |
| Treatment due to recurrence  surgery only  surgery + chemotherapy  chemotherapy only  unknown | 8 (11.9%)  5 (7.5%)  5 (7.5%)  8 (11.9%) |
| Chemotherapy regimens used  fluorouracil-based  kinase inhibitors-based  kinase inhibitors + fluorouracil-based | 3 (23.1%)  6 (46.2%)  4 (30.8%) |

Abbreviations: NAFLD, non-alcoholic fatty liver disease

**Table S2.** Histopathological features of hepatocellular carcinoma

| **Variables** |  |
| --- | --- |
| Tumor grade, Edmondson-Steiner, n (%)  1  2  3  4  Tumor grade, WHO, n (%)  1  2  3  4 | 0 (0%)  47 (70.1%)  18 (26.9%)  2 (3.0%)  32 (47.8%)  33 (49.3%)  2 (3.0%)  0 (0%) |
| Growth pattern, n (%)  desmoplastic  infiltrative  pushing  mixed (desmoplastic, infiltrative, pushing) | 31 (46.3%)  2 (3.0%)  0 (0%)  34 (50.7%) |
| Encapsulation (0/1/2/3), n (%) | 1/9/26/31 (1.5%/13.4%/38.8%/46.3%) |
| Amount of stromal component, n (%)  0  1  2  3 | 14 (20.9%)  27 (40.3%)  14 (20.9%)  12 (17.9%) |
| Cytological variants, n (%)  Typical (hepatocyte-like)  Clear cells  Steatohepatitis-like  Mixed | 48 (71.6%)  4 (6.0%)  3 (4.5%)  12 (17.9%) |
| Architectural grade, n (%)  Microtrabecular, grade I  Pseudoglandular, grade II  Midtrabecular, grade III  Macrotrabecular and solid/bizarre, grade IV | 16 (23.9%)  15 (22.4%)  28 (41.8%)  8 (11.9%) |
| Nuclear grade, n (%)  I  II  III  IV | 18 (26.9%)  34 (50.7%)  14 (20.9%)  1 (1.5%) |
| Nucleolar grade, n (%)  I  II  III  IV | 17 (25.4%)  25 (31.3%)  25 (31.3%)  0 (0%) |
| Tumor micronodularity, yes/no, n (%) | 28/49 (41.8%/58.2%) |
| Microvascular invasion, yes/no, n (%) | 18/49 (26.9%/43.1%) |
| Microsatellites, yes/no, n (%) | 30/37 (44.8%/55.2%) |
| Tumor necrosis, yes/no, n (%) | 27/50 (40.3%/59.7%) |

**Table S3.** Antibodies and detection systems used in immunohistochemical staining

| **Marker** | **Identified cell type** | **Primary antibody** | **Detection Kit** |
| --- | --- | --- | --- |
| **CD68** | pan-macrophages | BOND™ Ready-To-Use Primary Antibody, CD68 (514H12), Catalog No: PA0273 | BOND Polymer Refine Detection |
| **CD163** | Kupffer cells, M2 macrophages | BOND™ Ready-To-Use Primary Antibody, CD163 (10D6), Catalog No: PA0090 | BOND Polymer Refine Detection |
| **CD117** | mast cells | BOND™ Ready-To-Use Primary Antibody, CD117 (EP10), Catalog No: PA0007 | BOND Polymer Refine Detection |

**Table S4.** The estimated probability of outcomes in Kaplan-Meier analysis

|  | 1 year | 3 years | 5 years |
| --- | --- | --- | --- |
| RFP | 74.6% (63.6%-85.6%) | 55.9 % (41.9%-69.9%) | 48.7% (33.2%-64.4%) |
| DFS | 67.6% (56.1%-79.1%) | 43.2 % (30.1%-56.2%) | 33.2% (20.0%-46.5%) |
| OS | 88.1% (80.1%-96.1%) | 70.6 % (58.5%-82.6%) | 49.4% (35.1%-63.6%) |

Abbreviations: RFP, recurrence-free proportion; DFS, disease-free survival; OS, overall survival

**Table S5.** Correlation between area fraction of CD68+ and CD163+ macrophages and CD117+ mast cells in different ROIs (Spearman ρ)

| **CD68+ cells** | | | |
| --- | --- | --- | --- |
|  | IM | OM | PT, n=63 |
| TC, n=66 | ρ=0.77, P<0.001 | ρ=0.47, P<0.001 | ρ=0.47, P<0.001 |
| IM, n=66 |  | ρ=0.62, P<0.001 | ρ=0.46, P<0.001 |
| OM, n=66 |  |  | ρ=0.80, P<0.001 |
| **CD163+ cells** | | | |
|  | IM | OM | PT, n=64 |
| TC, n=65 | ρ=0.74, P<0.001 | ρ=0.33, P<0.001 | ρ=0.22, P<0.001 |
| IM, n=65 |  | ρ=0.47, P<0.001 | ρ=0.43, P<0.001 |
| OM, n=65 |  |  | ρ=0.84, P<0.001 |
| **CD117+ cells** | | | |
|  | IM | OM | PT, n=64 |
| TC, n=65 | ρ=0.59, P<0.001 | ρ=0.36, P=0.003 | ρ=0.26, P=0.037 |
| IM, n=65 |  | ρ=0.55, P<0.001 | ρ=0.33, P=0.007 |
| OM, n=65 |  |  | ρ=0.73, P<0.001 |

Abbreviations: TC, tumor center; IM, inner margin; OM, outer margin; PT, peritumor area

**Table S6.** Significant associations between AF of CD68+ and CD163+ macrophages and CD117+ mast cells and clinical and pathology variables

|  | **TC, n=67** | | | **IM, n=67** | | | **OM, n=67** | | **PT, n=66** | | |
| --- | --- | --- | --- | --- | --- | --- | --- | --- | --- | --- | --- |
|  | CD68 | CD163 | CD117 | CD68 | CD163 | CD117 | CD68 | CD163 | CD68 | CD163 | CD117 |
| Tumor grade WHO, n=67 |  | 0.25* |  | 0.28 |  |  |  |  |  |  |  |
| Nuclear grade,  n=67 |  |  |  | 0.32 |  |  |  |  |  |  |  |
| Amount of stromal component, n=67 | 0.26 | 0.25 | 0.34 | 0.25 |  |  | 0.4 | 0.34 | 0.28 |  |  |
| Growth pattern,  n=67 |  |  |  |  |  |  | 0.39 | 0.46 | 0.31 | 0.43 |  |
| Nucleolargrade, n=67 |  | 0.33 |  |  |  | -0.28 |  |  |  |  |  |
| Age,  n=67 |  |  |  |  | 0.31 |  |  |  |  |  |  |

1. *: Spearman ρ, P<0.05.
2. Abbreviations: AF, area fraction; TC, tumor center; IM, inner margin; OM, outer margin; PT, peritumor area

**Table S7.** Significant differences in AF of CD68+ and CD163+ macrophages and CD117+ mast cells between patients with and without microsatellites and multiple nodules

|  | **TC, n=67** | | | **IM, n=67** | | | **OM, n=67** | | | **PT, n=66** | | |
| --- | --- | --- | --- | --- | --- | --- | --- | --- | --- | --- | --- | --- |
|  | CD68 | CD163 | CD117 | CD68 | CD163 | CD117 | CD68 | CD163 | CD117 | CD68 | CD163 | CD117 |
| Microsatellites,  yes (n=24) vs no (n=35) |  |  |  |  |  |  | 0.008 | 0.004 |  |  | 0.021 |  |
| Tumor micronodularity,  yes (n=27) vs no (n=39) |  |  | 0.026 |  |  |  | 0.048 | 0.048 |  | 0.008 |  |  |

a. P value of Mann-Whitney test. b. Abbreviations: TC, tumor center; IM, inner margin; OM, outer margin; PT, peritumor area

**Table S8.** Univariable analysis of clinical and pathology variables associated with time to recurrence (TTR), disease-free survival (DFS) and overall survival (OS)

|  | **TTR** | | | **DFS** | | | **OS** | | |
| --- | --- | --- | --- | --- | --- | --- | --- | --- | --- |
|  | HR | 95% CI | P | HR | 95% CI | P | HR | 95% CI | P |
| Growth type, n=67,  desmoplastic vs other | 1.08 | 0.51-2.30 | 0.835 | 1.15 | 0.64-2.07 | 0.646 | 1.58 | 0.82-3.05 | 0.176 |
| Extent of encapsulation, n=67  0-1  2 vs 0-1  3 vs 0-1 | 1.00  5.00  3.87 | 0.65-38.69  0.51-29.40 | 0.155*  0.122  0.191 | 1.00  1.59  1.09 | 0.59-4.30  0.41-2.90 | 0.439*  0.363  0.863 | 1.00  1.17 0.63 | 0.39-3.56  0.21-1.91 | 0.204*    0.779 0.412 |
| Microvascular invasion, n=67 (yes vs no) | 2.06 | 0.96-4.40 | 0.063 | 1.59 | 0.85-2.97 | 0.146 | 0.86 | 0.42-1.78 | 0.689 |
| Diabetes mellitus, n=67 (yes vs no) | 2.07 | 0.98-4.39 | 0.058 | 1.66 | 0.91-3.01 | 0.097 | 0.89 | 0.46-1.73 | 0.735 |
| Viral hepatitis, n=67 (yes vs no) | 0.66 | 0.19-2.26 | 0.505 | 0.46 | 0.16-1.31 | 0.145 | 0.49 | 0.15-1.62 | 0.244 |
| Gender, n=67 (males vs females) | 0.89 | 0.36-2.21 | 0.807 | 0.86 | 0.42-1.74 | 0.672 | 0.87 | 0.40-1.91 | 0.727 |
| Age, n=67 (continuous) | 0.96 | 0.93-0.99 | **0.007** | 0.98 | 0.95-1.01 | 0.106 | 1.02 | 0.99-1.06 | 0.254 |
| Tumor Size, n=67 (continuous) | 0.76 | 0.42-1.41 | 0.387 | 0.85 | 0.53-1.36 | 0.489 | 1.00 | 0.99-1.00 | 0.283 |
| Tumor grade (WHO) n=67  1  2-3 | 1.00  0.70 | 0.33-1.48 | 0.355 | 1.00  0.67 | 0.37-1.20 | 0.172 | 1.00  0.95 | 0.50-1.83 | 0.882 |
| Tumor grade (Edmondson-Steiner), n=67  2  3-4 | 1.00  0.58 | 0.23-1.43 | 0.212 | 1.00  0.72 | 0.38-1.39 | 0.313 | 1.00  1.27 | 0.65-2.50 | 0.487 |
| Amount of stromal component, n=67  0  1  2  3 | 1.00  0.44  0.48  1.04 | 0.17-1.16  0.15-1.54  0.35-3.14 | 0.226*  0.096  0.218  0.939 | 1.00  0.53  0.66  1.00 | 0.24-1.17  0.27-1.62  0.39-2.58 | 0.331*  0.116  0.367  0.996 | 1.00  0.74  1.05  1.60 | 0.31-1.79  0.40-2.75  0.56-4.52 | 0.455*  0.508  0.924  0.380 |
| Nuclear grade, n=67  I  II  III-IV | 1.00  0.69  0.41 | 0.30-1.57  0.14-1.20 | 0.240*  0.378  0.105 | 1.00  0.70  0.50 | 0.36-1.37  0.23-1.11 | 0.224*  0.298  0.088 | 1.00  1.41  1.25 | 0.65-3.08  0.55-2.84 | 0.685*  0.389  0.596 |
| Nucleolar grade, n=67  I  II  III | 1.00  0.47  0.70 | 0.19-1.20  0.29-1.68 | 0.288*  0.116  0.420 | 1.00  0.53  0.80 | 0.25-1.12  0.40-1.62 | 0.230*  0.098  0.536 | 1.00  1.10  1.70 | 0.48-2.51  0.75-3.85 | 0.369*  0.824  0.204 |
| Necrosis, n=67 (yes vs no) | 0.88 | 0.39-1.96 | 0.752 | 1.25 | 0.69-2.26 | 0.464 | 1.86 | 0.97-3.56 | 0.063 |
| Tumor micronodularity, n=67 (yes vs no) | 0.87 | 0.40-1.91 | 0.736 | 1.04 | 0.57-1.90 | 0.888 | 1.48 | 0.77-2.86 | 0.237 |
| Microsatellites, n=67 (yes vs no) | 1.09 | 0.50-2.35 | 0.831 | 1.37 | 0.76-2.47 | 0.303 | 1.28 | 0.67-2.45 | 0.461 |

**Table S8 (continued).** Univariable analysis of clinical and pathology variables associated with time to recurrence (TTR), disease-free survival (DFS) and overall survival (OS)

|  | **TTR** | | | **DFS** | | | **OS** | | |
| --- | --- | --- | --- | --- | --- | --- | --- | --- | --- |
|  | HR | 95% CI | P | HR | 95% CI | P | HR | 95% CI | P |
| TNM stage, n=67  1  2  3-4 | 1.00  1.37  2.51 | 0.53-3.50  0.91-6.93 | 0.245*  0.517  0.075 | 1.00  1.00  1.70 | 0.47-2.14  0.70-4.11 | 0.994  0.243 | 1.00  1.13  0.71 | 0.51-2.51  0.21-2.34 | 0.787*  0.765  0.569 |
| Alpha-fetoprotein, n=46 (continuous) | 1.00 | 1.00-1.00 | 0.880 | 1.00 | 1.00-1.00 | 0.408 | 1.00 | 0.99-1.00 | 0.116 |
| Child-Pugh score, n=65 (A vs B) | 1.19 | 0.16-8.86 | 0.867 | 1.81 | 0.55-5.95 | 0.332 | 2.95 | 0.89-9.79 | 0.077 |

a. “No”, female gender, “Child-Pugh A score”, desmoplastic growth type, or the lowest category were the reference categories for dichotomous variables.

b. HR is for each one-unit change in the continuous predictor variables. Bold values indicate statistical significance at the P <0.05 level.

c. * - P for type 3 Wald test.

d. Since majority of patients received chemotherapy due to recurrence, testing that factor as a predictor for DFS or OS would generate a bias by indication.

Abbreviations: HR, hazard ratio; CI, confidence interval; TTR, time to recurrence; DFS, disease-free survival

**Table S9.** Univariable analysis of clinical and pathology variables associated with time to recurrence (TTR), disease-free survival (DFS) and overall survival (OS) in 58 non-viral HCC patients.

|  | **TTR** | | | **DFS** | | | **os** | | |
| --- | --- | --- | --- | --- | --- | --- | --- | --- | --- |
|  | HR | 95% CI | P | HR | 95% CI | P | HR | 95% CI | P |
| Growth type, n=58,  desmoplastic vs other | 0.93 | 0.43-2.06 | 0.866 | 1.03 | 0.55-1.90 | 0.937 | 1.45 | 0.72-2.90 | 0.295 |
| Extent of encapsulation, n=58  1  2 vs 1  3 vs 1 | 1.00  5.35  4.27 | 0.69-41.22  0.56-32.90 | 0.125*  0.108  0.163 | 1.00  1.55  1.34 | 0.57-4.24  0.50-3.60 | 0.673  0.393  0.562 | 1.00  1.07  0.73 | 0.35-3.27  0.24-2.23 | 0.573*  0.912  0.575 |
| Microvascular invasion, n=58 (yes vs no) | 1.94 | 0.86-4.40 | 0.113 | 1.61 | 0.83-3.14 | 0.159 | 1.00 | 0.47-2.16 | 0.99 |
| Diabetes mellitus, n=58 (yes vs no) | 2.19 | 0.99-4.84 | 0.054 | 1.81 | 0.97-3.38 | 0.063 | 1.05 | 0.53-2.09 | 0.892 |
| Gender, n=58 (males vs females) | 0.92 | 0.37-2.30 | 0.856 | 1.08 | 0.51-2.27 | 0.843 | 1.13 | 0.49-2.62 | 0.77 |
| Age, n=58 (continuous) | 0.96 | 0.93-0.99 | **0.003** | 0.97 | 0.94-0.99 | **0.016** | 1.02 | 0.98-1.05 | 0.339 |
| Tumor size, n=58 (continuous) | 1.00 | 0.99-1.01 | 0.711 | 1.00 | 0.99-1.01 | 0.733 | 0.99 | 0.99-1.00 | 0.170 |
| Tumor grade (WHO) n=58  1  2-3 | 1.00  0.76 | 0.35-1.67 | 0.494 | 1.00  0.64 | 0.34-1.20 | 0.167 | 1.00  0.88 | 0.44-1.75 | 0.710 |
| Tumor grade (Edmondson-Steiner), n=58  2  3-4 | 1.00  0.53 | 0.20-1.42 | 0.208 | 1.00  0.55 | 0.27-1.15 | 0.112 | 1.00  1.04 | 0.50-2.13 | 0.926 |
| Architectural grade, n=58  I  II  III  IV | 1.00  1.46  1.64  1.07 | 0.44-4.83  0.60-4.47  0.27-4.30 | 0.764*  0.537  0.334  0.923 | 1.00  1.07  1.05  0.80 | 0.42-2.72  0.50-2.21  0.30-2.12 | 0.949*  0.883  0.899  0.659 | 1.00  0.55  0.71  0.90 | 0.20-1.56  0.31-1.62  0.33-2.67 | 0.665*  0.263  0.414  0.903 |
| Stromal component, n=58  0  1  2  3 | 1.00  0.22  0.29  0.61 | 0.08-0.65  0.09-0.93  0.20-1.84 | 0.037*  **0.006**  **0.037**  0.380 | 1.00  0.24  0.26  0.46 | 0.10-0.56  0.10-0.68  0.18-1.21 | 0.012*  **0.001**  **0.006**  0.116 | 1.00  0.61  0.69  1.22 | 0.24-1.58  0.25-1.92  0.42-3.51 | 0.489*  0.311  0.478  0.716 |
| Nuclear grade, n=58  I  II  III- IV | 1.00  0.76  0.38 | 0.32-1.80  0.12-1.22 | 0.224*  0.538  0.104 | 1.00  0.71  0.42 | 0.35-1.44  0.17-0.99 | 0.112*  0.341  0.047 | 1.00  1.30  1.07 | 0.57-3.00  0.46-2.48 | 0.815*  0.533  0.874 |
| Nucleolar grade, n=58  I  II  III | 1.00  0.56  0.63 | 0.22-1.45  0.24-1.65 | 0.462*  0.231  0.347 | 1.00  0.57  0.61 | 0.26-1.23  0.31-1.44 | 0.353*  0.149  0.305 | 1.00  0.99  1.33 | 0.43-2.31  0.57-3.10 | 0.732*  0.985  0.513 |
| Necrosis, n=58 (yes vs no) | 0.85 | 0.36-2.00 | 0.712 | 1.11 | 0.60-2.09 | 0.736 | 1.59 | 0.80-3.15 | 0.188 |
| Tumor micronodularity, n=58 (yes vs no) | 0.76 | 0.33-1.72 | 0.507 | 0.95 | 0.50-1.79 | 0.861 | 1.35 | 0.67-2.74 | 0.407 |
| Microsatellites, n=58 (yes vs no) | 0.98 | 0.44-2.18 | 0.959 | 1.06 | 0.57-1.96 | 0.855 | 0.97 | 0.49-1.90 | 0.921 |
| TNM stage, n=58  1  2  3-4 | 1.00  1.74  2.48 | 0.66-4.56  0.88-6.96 | 0.208*  0.264  0.085 | 1.00  0.99  1.53 | 0.44-2.44  0.62-3.74 | 0.664*  0.989  0.354 | 1.00  1.04  0.62 | 0.46-2.37  0.19-2.09 | 0.722*  0.918  0.443 |
| Alpha-fetoprotein, n=37 (continuous) | 1.00 | 0.99-1.00 | 0.539 | 1.00 | 0.99-1.00 | 0.627 | 1.00 | 0.99-1.00 | 0.156 |
| Child-Pugh score, n=56 (A vs B) | 1.03 | 0.14-7.75 | 0.978 | 0.95 | 0.22-4.03 | 0.939 | 1.77 | 0.42-7.52 | 0.439 |

1. No”, female gender, “Child-Pugh A score”, desmoplastic growth type, or the lowest category were the reference categories for dichotomous variables.
2. b. HR is for each one-unit change in the continuous predictor variables.
3. Bold values indicate statistical significance at the P <0.05 level.
4. * - P for type 3 Wald test.

Abbreviations: HR, hazard ratio; CI, confidence interval; TTR, time to recurrence; DFS, disease-free survival

**Table S10.** Area fraction of tumor-infiltrating macrophages and mast cells per individual ROI associated with TTR, DFS, and OS (univariable analysis) in 58 non-viral HCC patients

| Cell types | **TTR** | | | **DFS** | | | **OS** | | |
| --- | --- | --- | --- | --- | --- | --- | --- | --- | --- |
|  | HR | 95% CI | P | HR | 95% CI | P | HR | 95% CI | P |
| Tumor center | | | | | | | | | |
| CD68 | 0.41 | 0.18 -0.91 | **0.030** | 0.48 | 0.25-0.92 | **0.026** | 1.02 | 0.47 - 2.19 | 0.970 |
| CD163 | 0.54 | 0.23 -1.27 | 0.160 | 0.57 | 0.29 -1.13 | 0.108 | 1.07 | 0.48 -2.38 | 0.880 |
| CD117 | 0.50 | 0.20-1.23 | 0.130 | 0.46 | 0.21-0.99 | **0.046** | 0.89 | 0.37-2.17 | 0.800 |
| Inner margin | | | | | | | | | |
| CD68 | 0.47 | 0.19 -1.13 | **0.090** | 0.43 | 0.21 -0.87 | **0.020** | 0.71 | 0.32 -1.60 | 0.410 |
| CD163 | 0.68 | 0.29 -1.59 | 0.380 | 0.98 | 0.48 -2.02 | 0.959 | 1.68 | 0.75 -3.80 | 0.210 |
| CD117 | 0.46 | 0.20-1.09 | 0.080 | 0.46 | 0.23 -0.91 | **0.027** | 0.37 | 0.17-0.77 | **0.010** |
| Outer margin | | | | | | | | | |
| CD68 | 0.63 | 0.25-1.60 | 0.340 | 0.57 | 0.27 -1.15 | 0.114 | 0.76 | 0.34-1.69 | 0.500 |
| CD163 | 1.20 | 0.44 -3.25 | 0.720 | 1.14 | 0.77 -1.69 | 0.511 | 1.36 | 0.56 -3.30 | 0.500 |
| CD117 | 1.23 | 0.46-3.30 | 0.680 | 0.97 | 0.46-2.06 | 0.935 | 1.10 | 0.48-2.54 | 0.830 |
| Peritumor area | | | | | | | | | |
| CD68 | 0.95 | 0.35-2.58 | 0.920 | 1.10 | 0.50-2.42 | 0.821 | 1.01 | 0.45 -2.297 | 0.970 |
| CD163 | 2.33 | 0.76-7.15 | 0.140 | 1.68 | 1.09-2.58 | **0.019** | 1.20 | 0.56 -2.56 | 0.650 |
| CD117 | 0.58 | 0.25-1.36 | 0.210 | 0.57 | 0.29-1.15 | 0.117 | 0.70 | 0.33 -1.47 | 0.350 |

1. For all cells and regions of interest, the area fraction of TAMs and mast cells per area section (mm^2^) were converted into percentiles and then categorized into low (0-25 percentile) vs high (25-100 percentile). Hazard ratios show the relative risk compared with 1 for the low AF. Bold cells indicate statistical significance at the P <0.05 level.
2. Abbreviations: HR, hazard ratio; CI, confidence interval; TTR, time to recurrence; DFS, disease-free survival; OS, overall survival

**Table S11.** Area fraction of tumor-infiltrating macrophages and mast cells per individual ROI associated with DFS and OS (univariable analysis) in 54 patients not treated with chemotherapy

| Cell types | **DFS** | | | **OS** | | |
| --- | --- | --- | --- | --- | --- | --- |
|  | HR | 95% CI | P- value | HR | 95% CI | P |
| CD68 TC | 0.38 | 0.18-0.78 | **0.009** | 1.09 | 0.46-2.60 | 0.843 |
| CD68 IM | 0.34 | 0.16-0.71 | **0.004** | 0.79 | 0.33-1.90 | 0.605 |
| CD68 OM | 0.74 | 0. 32-1.72 | 0.484 | 0.82 | 0.33-2.06 | 0.676 |
| CD68 PT | 1.16 | 0.53-2.55 | 0.716 | 1.08 | 0.47-2.51 | 0.852 |
| CD163 TC | 0.79 | 0.38-1.64 | 0.524 | 1.38 | 0.58-3.30 | 0.468 |
| CD163 IM | 1.13 | 0.52-2.46 | 0.751 | 1.37 | 0.59-3.14 | 0.464 |
| Cd163 OM | 2.04 | 0.83-5.00 | 0.118 | 1.31 | 0.55-3.12 | 0.536 |
| CD163 PT | 3.20 | 1.24-8.29 | **0.017** | 1.00 | 0.46-2.21 | 0.992 |
| Cd117 TC | 0.51 | 0.24-1.09 | 0.084 | 1.30 | 0.52-3.23 | 0.572 |
| CD117 IM | 0.39 | 0.17-0.88 | **0.024** | 0.26 | 0.10-0.66 | **0.004** |
| Cd117 OM | 0.71 | 0.30-1.69 | 0.442 | 0.77 | 0.31-1.92 | 0.572 |
| CD117 PT | 0.36 | 0.16-0.78 | **0.009** | 0.49 | 0.22-1.12 | 0.090 |

1. For all cells and regions of interest, the area fraction of TAMs and mast cells per area section (mm2) were converted into percentiles and then categorized into low (0-25 percentile) vs high (25-100 percentile). Hazard ratios show the relative risk compared with 1 for the low AF. Bold cells indicate statistical significance at the P <0.05 level.
2. Abbreviations: HR, hazard ratio; CI, confidence interval; DFS, disease-free survival; OS, overall survival, TC, tumor center; IM, inner margin; OM, outer margin; PT, peritumor area

**Table S12.** Multivariable Cox-regression for DFS adjusted for age and amount of stromal component in 58 non-viral HCC patients

| Cell types | HR | 95% CI | P |
| --- | --- | --- | --- |
| CD68 TC | Not in the model |  |  |
| age | 0.97 | 0.93-1.00 | **0.039** |
| Stroma  1  2  3 | 0.28  0.24  0.46 | 0.12-0.68  0.09-0.63  0.18-1.20 | **0.005**  **0.004**  0.112 |
|  |  |  |  |
| CD68 IM | Not in the model |  |  |
| age | 0.97 | 0.94-1.00 | **0.045** |
| Stroma  1  2  3 | 0.29  0.25  0.48 | 0.12-0.71  0.09-0.67  0.18-1.27 | **0.007**  **0.006**  0.139 |
|  |  |  |  |
| CD117 TC | Not in the model |  |  |
| age | 0.97 | 0.93-1.00 | **0.039** |
| Stroma  1  2  3 | 0.28  0.24  0.46 | 0.12-0.68  0.09-0.63  0.18-1.20 | **0.005**  **0.004**  0.112 |
|  |  |  |  |
| CD117 IM | **0.39** | 0.19-0.82 | **0.013** |
| age | 0.96 | 0.93-1.00 | **0.022** |
| Stroma  1  2  3 | 0.27  0.26  0.40 | 0.11-0.65  0.10-0.72  0.15-1.07 | **0.004**  **0.010**  0.069 |
|  |  |  |  |
| CD163 PT | Not in the model |  |  |
| age | 0.96 | 0.93-0.99 | **0.020** |
| Stroma  1  2  3 | 0.29  0.20  0.40 | 0.12-0.70  0.07-0.56  0.15-1.07 | **0.006**  **0.002**  0.069 |

1. Hazard ratios show the relative risk compared with 1 for the low AF. Bold cells indicate statistical significance at the P <0.05 level.
2. Abbreviations: HR, hazard ratio; CI, confidence interval; DFS, disease-free survival; TC, tumor center; IM, inner margin; PT, peritumor area

**Table S13**. Area fraction of combinations between tumor-infiltrating CD68+ and CD163+ macrophages and CD117+ mast cells associated with TTR, DFS, and OS (univariable analysis) in 67 patients

| Cell types | **DFS** |  |  | **TTR** |  |  | **OS** |  |  |
| --- | --- | --- | --- | --- | --- | --- | --- | --- | --- |
|  | HR | 95% CI | P | HR | 95% CI | P | HR | 95% CI | P |
|  |  |  | 0.040* |  |  | 0.093* |  |  | 0.32* |
| CD68 TC low CD163 TC low | 1.00 |  |  | 1.00 |  |  | 1.00 |  |  |
| CD68 TC low CD163 TC high | 2.61 | (0.92-7.40) | 0.071 | 2.20 | (0.63-7.67) | 0.216 | 3.40 | (1.00-12.0) | 0.057 |
| CD68 TC high CD163 TC low | 0.55 | (0.18-1.63) | 0.278 | 0.70 | (0.20-2.40) | 0.568 | 1.62 | (0.50-5.33) | 0.426 |
| CD68 TC high CD163 TC high | 0.63 | (0.30-1.32) | 0.216 | 0.48 | (0.19-1.22) | 0.123 | 1.71 | (0.63-4.60) | 0.291 |
|  |  |  | 0.029* |  |  | 0.279* |  |  | 0.496* |
| CD68 IM low CD163 IM low | 1.00 |  |  | 1.00 |  |  | 1.00 |  |  |
| CD68 IM low CD163 IM high | 0.71 | (0.24-2.09) | 0.537 | 0.68 | (0.18-2.58) | 0.57 | 1.06 | (0.29-3.85) | 0.926 |
| CD68 IM high CD163 IM low | 0.21 | (0.07-0.65) | **0.007** | 0.34 | (0.09-1.24) | 0.102 | 0.57 | (0.17-1.91) | 0.363 |
| CD68 IM high CD163 IM high | 0.43 | (0.17-1.05) | 0.064 | 0.36 | (0.12-1.12) | 0.076 | 1.03 | (0.35-3.07) | 0.959 |
|  |  |  | 0.033* |  |  | 0.165* |  |  | 0.392* |
| CD68 TC low CD163 PT low | 1.00 |  |  | 1.00 |  |  | 1.00 |  |  |
| CD68 TC low CD163 PT high | 1.34 | (0.41-4.38) | 0.629 | 1.41 | (0.29-7.00) | 0.673 | 0.39 | (0.10-1.49) | 0.167 |
| CD68 TC high CD163 PT low | 0.33 | (0.09-1.19) | 0.089 | 0.37 | (0.07-2.10) | 0.261 | 0.53 | (0.16-1.72) | 0.290 |
| CD68 TC high CD163 PT high | 0.89 | (0.29-2.68) | 0.830 | 0.77 | (0.17-3.58) | 0.742 | 0.80 | (0.26-2.44) | 0.690 |
|  |  |  | 0.006* |  |  | 0.026* |  |  | 0.572* |
| CD68 IM low CD163 PT low | 1.00 |  |  | 1.00 |  |  | 1.00 |  |  |
| CD68 IM low CD163 PT high | 0.64 | (0.19-2.12) | 0.465 | 0.42 | (0.10-1.76) | 0.234 | 0.44 | (0.12-1.60) | 0.212 |
| CD68 IM high CD163 PT low | 0.15 | (0.04-0.54) | **0.004** | 0.09 | (0.02-0.48) | **0.005** | 0.42 | (0.13-1.34) | 0.142 |
| CD68 IM high CD163 PT high | 0.45 | (0.15-1.36) | 0.159 | 0.33 | (0.09-1.19) | 0.089 | 0.53 | (0.17-1.63) | 0.267 |
|  |  |  | 0.011* |  |  | 0.129* |  |  | 0.002* |
| CD68 IM low CD117 IM low | 1.00 |  |  | 1.00 |  |  | 1.00 |  |  |
| CD68 IM low CD117 IM high | 0.30 | (0.10-0.93) | **0.037** | 0.40 | (0.10-1.63) | 0.199 | 0.07 | (0.02-0.28) | **0.001** |
| CD68 IM high CD117 IM low | 0.26 | (0.08-0.83) | **0.024** | 0.28 | (0.06-1.25) | 0.095 | 0.19 | (0.05-0.66) | **0.009** |
| CD68 IM high CD117 IM high | 0.15 | (0.05-0.42) | **0.001** | 0.20 | (0.05-0.71) | **0.013** | 0.10 | (0.03-0.29) | **0.001** |
|  |  |  | 0.017* |  |  | 0.092* |  |  | 0.38* |
| CD68 TC low CD117 PT low | 1.00 |  |  | 1.00 |  |  | 1.00 |  |  |
| CD68 TC low CD117 PT high | 0.32 | (0.11-0.95) | **0.039** | 0.41 | (0.11-1.52) | 0.183 | 0.36 | (0.10-1.38) | 0.137 |
| CD68 TC high CD117 PT low | 0.30 | (0.10-0.91) | **0.033** | 0.29 | (0.07-1.20) | 0.089 | 0.81 | (0.23-2.84) | 0.740 |
| CD68 TC high CD117 PT high | 0.18 | (0. 07-0.47) | **0.001** | 0.20 | (0.06-0.65) | 0.008 | 0.52 | (0.17-1.56) | 0.243 |

**Table S13 (continued)**. Area fraction of combinations between tumor-infiltrating CD68+ and CD163+ macrophages and CD117+ mast cells associated with TTR, DFS, and OS (univariable analysis) in 67 patients

| Cell types | **DFS** | | | **TTR** | | | **OS** | | |
| --- | --- | --- | --- | --- | --- | --- | --- | --- | --- |
|  | HR | 95% CI | P | HR | 95% CI | P | HR | 95% CI | P |
|  |  |  | 0.014* |  |  | 0.165* |  |  | 0.362* |
| CD68 IM low CD117 PT low | 1.00 |  |  | 1.00 |  |  | 1.00 |  |  |
| CD68 IM low CD117 PT high | 0.34 | (0.11-1.07) | 0.064 | 0.50 | (0.12-2.12) | 0.348 | 0.37 | (0.10-1.42) | 0.148 |
| CD68 IM high CD117 PT low | 0.28 | (0.09-0.87) | **0.027** | 0.39 | (0.09-1.62) | 0.193 | 0.60 | (0.18-2.03) | 0.411 |
| CD68 IM high CD117 PT high | 0.19 | (0.07-0.47) | **0.001** | 0.25 | 0.08-0.81) | 0.021 | 0.41 | (0.15-1.11) | 0.078 |
|  |  |  | 0.025* |  |  | 0.275* |  |  | 0.252* |
| CD163 PT low CD117 PT low | 1.00 |  |  | 1.00 |  |  | 1.00 |  |  |
| CD163 PT low CD117 PT high | 0.19 | (0.05-0.84) | **0.028** | 0.33 | (0.03-3.29) | 0.346 | 0.28 | (0.07-1.08) | 0.064 |
| CD163 PT high CD117 PT low | 0.94 | (0.24-3.64) | 0.923 | 1.30 | (0.15-11.06) | 0.808 | 0.50 | (0.13-1.99) | 0.328 |
| CD163 PT high CD117 PT high | 0.55 | (0.16-1.89) | 0.340 | 0.80 | (0.10-6.19) | 0.834 | 0.29 | (0.08-1.02) | 0.054 |
|  |  |  | 0.017* |  |  | 0.338* |  |  | 0.045* |
| CD163 PT low CD117 IM low | 1.00 |  |  | 1.00 |  |  | 1.00 |  |  |
| CD163 PT high CD117 IM low | 0.90 | (0.23-3.52) | 0.874 | 1.55 | (0.18-13.17) | 0.689 | 0.65 | (0.17-2.45) | 0.522 |
| CD163 PT low CD117 IM high | 0.19 | (0.04-0.78) | **0.021** | 0.52 | (0.06-4.42) | 0.548 | 0.26 | (0.07-0.95) | **0.042** |
| CD163 PT high CD117 IM high | 0.54 | (0.16-1.86) | 0.332 | 0.93 | (0.12-7.22) | 0.994 | 0.23 | (0.07-0.83) | **0.024** |

a. Hazard ratios show the relative risk compared with 1 for the low AF. Bold values indicate statistical significance at the P <0.05 level.

b. *Type 3 Wald test P value.

c. Abbreviations: HR, hazard ratio; CI, confidence interval; TTR, time to recurrence; DFS, disease-free survival; OS, overall survival; TC, tumor center; IM, inner margin; PT, peritumor area
